# Reactive phenolic solvents applied to the synthesis of renewable aromatic polyesters with high isosorbide content†

**DOI:** 10.1039/d2py01578a

**Published:** 2023-06-26

**Authors:** Bruno Bottega Pergher, Narcisa Girigan, Sietse Vlasblom, Daniel H. Weinland, Bing Wang, Robert-Jan van Putten, Gert-Jan M. Gruter

**Affiliations:** a Van't Hoff Institute of Molecular Sciences, University of Amsterdam P.O. Box 94720 1090GS Amsterdam The Netherlands g.j.m.gruter@uva.nl; b Avantium Chemicals BV Zekeringstraat 29 1014BV Amsterdam The Netherlands

## Abstract

High boiling point phenolic reactive solvents like *p*-cresol could play a key role in improving the synthesis of aromatic polyesters with a high content of secondary diols such as isosorbide. Previously, our group showed that this method significantly improves the synthesis of poly(isosorbide succinate). In this work, terephthalic acid and 2,5-furandicarboxylic acid were used as building blocks for the synthesis of high *T*_g_ polyesters with high isosorbide content (>30 mol% of diols) and high molecular weight (*M*_n_ > 24 kg mol^−1^). A number of reactive and non-reactive solvents were tested in this work, and the results clearly point to a significant improvement when using reactive solvents, in terms of molecular weight and polycondensation time, especially for the case of *p*-cresol. The synthesis method was successfully scaled to 1 kg, showing promise for production at industrial scale. A method to remove these solvents (including end groups) from the polymers, which uses small excesses of isosorbide (1.5–3.0%) in the feed, is also presented.

## Abstract 2

Reactive phenolic solvents like *p*-cresol can be used to produce high MW polyesters (30–40 kDa) with high isosorbide content (50%). Aromatic polyesters like PICT can be made within very short polycondensation times (1–2 h).

## Graphical abstract

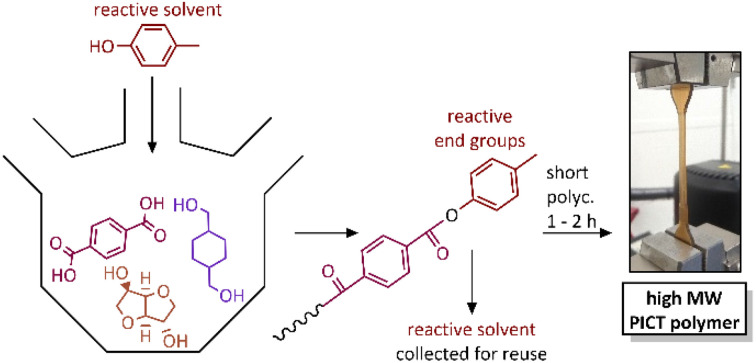

## Introduction

Currently about 3% of global greenhouse gas (GHG) emissions can be attributed to the production of plastics. The expected growth of the plastics industry and the targets for GHG emission reduction would result in 30% of all GHG emissions in 2050 to come from plastics production if the same types of plastics and resources are used.^1,2^ Knowing that for certain key industrial processes it is very difficult to switch to non-fossil energy/resources, this shows that the plastics industry must switch to different sources. Plastics production is expected to grow to around 1200 Mt per year in 2050 and in order to do this sustainably >60% should come from recycling, with the other sources being CO_2_ (∼25%) and biomass (10–15%).^3^ From both a (chemical) recycling and sourcing point of view polyesters make the most sense as plastics of the future.

Chemical recycling is possible due to the ease in cleaving the ester bonds, allowing relatively low temperatures with the use of nucleophilic solvents. A common example is the glycolysis of poly(ethylene terephthalate), PET, to obtain bis(2-hydroxyethyl)terephthalate, BHET, at temperatures around 200 °C.^4^ Once BHET or terephthalic acid is recovered, apart from direct use, it can be converted into 1,4-cyclohexanedimethanol (CHDM),^5^ a useful monomer for high impact and high glass transition temperature (*T*_g_) materials. The chemical recycling of other classes of polymers, however, may be significantly more difficult and energy-intensive. Such is the case of polyolefins, which typically undergo pyrolysis at temperatures above 500 °C to yield a mixture alkanes, alkenes and aromatics.^6^ Only relatively small amounts of the olefin building blocks (ethylene or propylene) can be resourced (closed-loop recycled) in this way, which means that large amounts of virgin, likely fossil, feedstock are always required.^7^ Alternatively, bio-based polyester monomers can be used, which include lactic acid from glucose, commercially used to produce poly(lactic acid) (PLA), 2,5-furandicarboxylic acid (FDCA) from fructose, used for the production of poly(ethylene furanoate) (PEF) and succinic acid, also from glucose, which can be used for producing poly(butylene succinate) (PBS). Furthermore, biomass (and CO_2_) are high in oxygen content and therefore are good starting materials from which to make atom efficient diacids and diols, for instance.

Most commercial polyesters cannot handle applications with high temperature exposure (≥100 °C; *e.g.* boiling water) while there is a relevant need for plastics with high glass transition temperatures. Examples of products that could benefit from this feature are plastic bottles to be hot-filled, washed with hot water for sanitation – enabling them to be reused, as well as cups to be made of (or coated with) biodegradable high-*T*_g_ plastics, for instance. A common bio-based co-monomer used to increase the *T*_g_ of plastics and make such applications possible is isosorbide (IS), though its incorporation in high amounts in polyesters has difficulties.

Since isosorbide is a secondary diol (both hydroxyl groups) and as it has one endo hydroxyl group, its reactivity is limited, which makes chain growth of polymers with high isosorbide content (>30 mol% relative to total diol) difficult. Additionally, the high temperatures and long reaction times needed for isosorbide to react, and the reversibility of the (trans)esterification reaction may cause it to leave the system (dragging), especially with high melting point oligomers/polymers, which may negatively affect the acid–alcohol ratio. To overcome that drawback, an excess of diols in relation to diacids is typically used, as the diols are volatile and can be removed during polycondensation at reduced pressure to bring the diacid–diol ratio (close) to 1 at the end of the polycondensation.

The literature provides examples of synthesis of terephthalic acid-based polyesters with isosorbide making use of a diol excess in the feed. Commonly, the ratio diol/diacid approaches 1.1–1.2.^8–10^ The diol excess seems to work by compensating the loss of less reactive diols like isosorbide and by reducing the viscosity of the mixture. The isosorbide content in the polymer still may not reach the targeted value, potentially leading to insufficient chain growth, in turn producing polymers with relatively low molecular weight (MW) and poor mechanical properties. Other methods include the use of terephthaloyl chloride as a more reactive acid substitute for terephthalic acid, or the use of macromonomers.^11,12^ Similar trends are seen for polyesters based on 2,5-furandicarboxylic acid (FDCA).^13–18^ As an alternative, we developed a synthesis method with reactive high boiling point phenolic solvents to circumvent such obstacles. Some of these compounds are known to be used in small amounts as radical inhibitors, stopping the formation of cross-links in polycondensations of acids like itaconic acid.^19,20^ We, however, use them in larger amounts to facilitate chain growth. Intuitively, it seems undesirable to add significant amounts of mono-functional alcohols as they are expected to act as a chain terminator. However, with specific alcohols this is not the case and instead of chain termination they rather act as a chain extender due to their outstanding leaving group ability.

Our group previously proved the effectiveness of phenolic solvents like *p*-cresol when developing polyesters like PISA, poly(isosorbide succinate).^21^ The solvents provide the (di)acids with reactive leaving groups, thus enhancing reactivity between isosorbide and the activated esters. The current work focuses on the exploration of the synthesis method with reactive phenolic solvents applied to the case of aromatic polyesters, namely poly(isosorbide-*co*-cyclohexanedimethanol)terephthalate *i.e.* PICT and poly(isosorbide-*co*-cyclohexanedimethanol)furanoate *i.e.* PICF.

## Experimental

### Materials

Terephthalic acid (purity ≥99%) was purchased from Acros Organics and butyltin hydroxide oxide hydrate (97%) from Sigma-Aldrich. 1,4-Cyclohexanedimethanol (>99%) with 70% of the *trans* isomer, dimethyl terephthalate (DMT, >99%) were purchased from TCI. Isosorbide was acquired from Roquette Frères (≥99.9%). 2,5-Furandicarboxylic acid (FDCA, >99.5%) was provided by Avantium.

*p*-Cresol (4-methyl phenol, >99%), guaiacol (2-methoxy phenol, >99%) and 4-chlorophenol (>99%) were purchased from ACROS Organics, 4-ethyl phenol (98%), 4-methoxyphenol (99%) and dimethoxybenzene (99%) from Sigma Aldrich and 4-ethyl guaiacol (4-ethyl-2-methoxyphenol, 97%) from ABCR.

### Polyester synthesis with reactive solvents

The example below describes the synthesis of the polyester of terephthalic acid, cyclohexanedimethanol and isosorbide, PICT. PICF was synthesized in an analogous manner, with the main differences indicated in Table 1. The method has been developed within our group and was first presented in the literature by Weinland *et al.*^21^

**Table 1:** Synthesis of PICT and PICF under different conditions, using typical two-step polycondensation routes, unreactive solvents and reactive solvents

Sample^a^	Solvent [equiv. *vs*. diacid]	*M*_n_ b (kg mol^−1^) [*Đ*]	*T*_g_ (°C)	Solvent EG/total EG^c^ before vacuum (mol%)	Polymer composition^c^ IS/CHDM (mol%)	Solvent's (or water's) p*K*_a_	*t*_est_/*t*_PC_ (h)	*T*_est_ (boiling point)/*T*_PC_ d (°C)
	**PICT: feed = 1 equivalent TPA + 0.5 IS + 0.5 CHDM (+excess IS)**							
PICT	No solvent	13.7 [2.4]	135.8	0/4.7	47.0/52.0	14.00 (water)	12.0/1.5	260/260–285
PICT_(IS+1.5%)_	No solvent, excess IS	14.1 [2.4]	135.0	0/5.1	45.4/50.3	14.00	14/2.0	260/260–285
PICT_(IS+3%)_	No solvent, excess IS	14.6 [2.5]	138.6	0/4.2	49.6/49.8	14.00	9.3/3.3	260/260–285
PICT_(IS+10%)_	No solvent, excess IS	16.7 [2.5]	141.3	0/11.3	49.8/48.9	14.00	10.0/1.5	260/260–285
PICT/DMB^1.2^	1,4-Dimethoxy benzene [1.2], non-reactive	15.3 [2.1]	140.4	0/2.6	48.4/50.4	14.00	20.5/1.0	260 (235)/260–285
PICT/DPE^1.2^	Di-phenyl ether [1.2], non-reactive	15.2 [2.3]	140.5	0/4.1	48.9/50.8	14.00	8.0/2.3	260 (258)/260–285
PICT/Gu^1.2^	Guaiacol [1.2]	12.9 [2.4]	134.1	2.5/20.1	47.6/50.3	9.98	40.0/2.5	240 (205)/260–285
PICT/4EG^1.2^	4-Ethyl guaiacol [1.2]	15.2 [2.3]	139.4	8.7/22.0	48.8/50.7	10.31	7.0/1.8	260 (236)/260–285
PICT/MP^1.2^	4-Methoxy phenol [1.2]	24.0 [2.3]	141.6	24.7/44.3	49.9/49.9	10.21	7.0/1.0	260 (243)/260–285
PICT/ClPh^1.2^	4-Chloro phenol [1.2]	24.7 [2.8]	144.6	13.9/25.8	49.9/50.0	9.24	10.5/1.0	260 (220)/260–285
PICT/EP^1.2^	4-Ethyl phenol [1.2]	26.2 [2.3]	144.3	19.6/36.0	50.0/49.8	10.00	10.0/1.0	260 (218)/260–285
PICT/Cre^1.2^	4-Methyl phenol [1.2]	41.9 [2.0]	147.1	19.6/34.7	50.1/49.9	10.14	16.7/1.0	240 (202)/260–285
	**PICF: feed = 1 equivalent FDCA + 0.5 IS + 0.5 CHDM**							
PICF	No solvent	12.1 [2.3]	118.3	0/9.7	46.5/50.1	14.00	6.5/1.5	230/240–265
PICF/MP^1.5^	4-Methoxy phenol [1.5]	23.5 [1.9]	126.4	18.9/32.9	50.2/50.2	10.21	7.0/0.5	230 (243)/240–265
PICF/Cre^1.2^	4-Methyl phenol [1.2]	37.0 [2.1]	128.7	16.3/30.9	50.3/49.9	10.14	6.5/1.5	230 (202)/240–275

a The solvent names, when used, are indicated after the forward slash “/”. The superscript numbers refer to equivalent of solvent used in comparison to the acid. The subscript refers to the diol excess (isosorbide), in mol% *versus* the total diol content, used in the feed of the referred reaction.

b Molecular weight measurements determined *via* GPC, using HFIP as solvent, with PMMA standards. *Đ* is the dispersity, defined by *M*_w_/*M*_n_.

c Molar content, measured by ^1^H-NMR, of end groups at the end of (trans)esterification step (before vacuum), and polymer composition measured in final polymer sample. Measures shown in mol percent related to the terephthalate or furanoate unit/peak (repeat unit). For the end groups of acids, no titration was conducted, so the acid EGs are not accounted for. The inherent difficulty to accurately determine the co-monomer content in the polymers through NMR should be noted, which justifies some of the unexpected values going slightly above 1 : 1 acid : diol ratio.

d Oil temperature shown for esterification (*T*_esterif._) and polycondensation (*T*_PC_), and in brackets the reflux temperature for the solvent (boiling point) at ambient pressure.

**Scheme 1:**
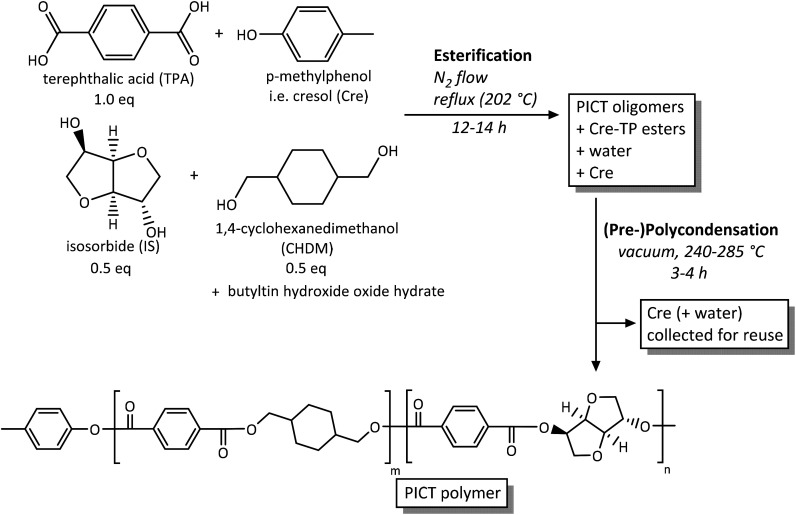
Synthesis of PICT using *p*-cresol as reactive solvent.

PICT was synthesized in a two-step melt polycondensation method, with high boiling point reactive solvents present from the start (Scheme 1), in three-necked round-bottom flasks of 100 mL. The basis for the experiments was the mass of diacid, typically 10 g. Total diols and diacid were fed at a 1.0 ratio (diols/diacid), except when explicitly stated otherwise (1.03, 1.05 or 1.10, using an excess of isosorbide). All components were weighed directly into the reaction flask or by using plastic trays: diols, diacid, solvent and catalyst (butyltin hydroxide oxide hydrate, 0.14 mol%) in the proportions described in Table 1. Once the vessel was set up with nitrogen gas flow (30–40 mL min^−1^) and a top mechanical stirrer, the heating was started, beginning the esterification step, at the oil bath temperatures listed in Table 1. Stirring started (100 rpm) after the solvent and diols or oligomer appeared to have melted or sufficiently dissolved. The conversion during this stage was tracked using ^1^H-NMR, by following the disappearance of unreacted IS and the formation of esters, combined with visually checking the homogeneity of the mixture, a result of the formation of terephthalate esters. Once esterification was deemed finished (end groups reached equilibrium), a second step was initiated under vacuum, typically after 8 to 14 hours.

The polycondensation step, in the same vessel and setup, was initiated by a gradual pressure reduction, starting at 400 mbar (pre-polycondensation) and decreasing to 0.3–1.0 mbar (polycondensation or “PC”). As viscosity increased, higher temperatures were applied in order to enable stirring and removal of the remainder of water and solvent (up to 285 °C for PICT). At this stage, at full vacuum, the polymerization was conducted until the viscosity of the melt prevented effective stirring, or else when no change could be noticed in torque or distillation temperature, typically after 1 to 2 hours of full vacuum (<1 mbar). For better comparison, however, samples were taken after the same amount of time, for instance for comparison of batches with reactive solvents against the other methods.

### Scaling up

The reactions conducted at larger scale were performed in a two-liter autoclave vessel from Büchi AG, type 3. The method employed is the same as described above, with differences in times and temperatures described in Table 3.

### Characterization

Molecular weight data were recorded using gel permeation chromatography (GPC) with a LC column from Agilent (Poroshell 120 EC-C18, 4.6 × 100 mm, 2.7 μm) calibrated with poly(methyl methacrylate) (PMMA) standards running with hexafluoroisopropanol (HFIP) as the eluent. The molecular weights are estimated from data obtained with a VWR Hitachi 5450 RI detector.

Proton nuclear magnetic resonance (^1^H-NMR) was performed on a Bruker AMX 400 (400 Hz) using as solvent deuterated chloroform (CDCl_3_) for pre-polymer samples and deuterated tetrachloroethane (TCE-d2) and CDCl_3_ for the polymer samples. The quantifications are made in relation to the repeat unit, which is assumed to be the diacid's integral. In the polymer, isosorbide was quantified with the peak between 4.96 and 5.05 ppm (2H), and CHDM with the peak between 1.00 and 1.20 ppm (4H, *trans*). The end groups were calculated as follows: for isosorbide, 1H around 4.90–4.95 ppm (*exo* hydroxyl) added to 2H around 3.82–3.86 ppm (*endo* hydroxyl); for CHDM, peak at 3.41 ppm for 2H (*trans*); for *p*-cresol and analogous to other phenolic solvent, the bound solvent can be identified around 7.2 ppm, in which each doublet represents 2H. Spectra with examples of end group calculations are shown in the ESI†

The glass transition temperatures were recorded with a differential scanning calorimeter DSC 3+ STARe from Mettler Toledo, using cycles at a rate of 10 °C min^−1^ for heating and −10 °C min^−1^ for cooling. The second heating cycle of each sample was used for determination of its glass transition temperature. The temperatures ranged from 25 °C to 300 °C, under nitrogen gas flow (50 mL min^−1^). *T*_m_ (melting) are not shown because all the polymers are amorphous.

## Results and discussion

### Polyester synthesis

**Fig. 1:**
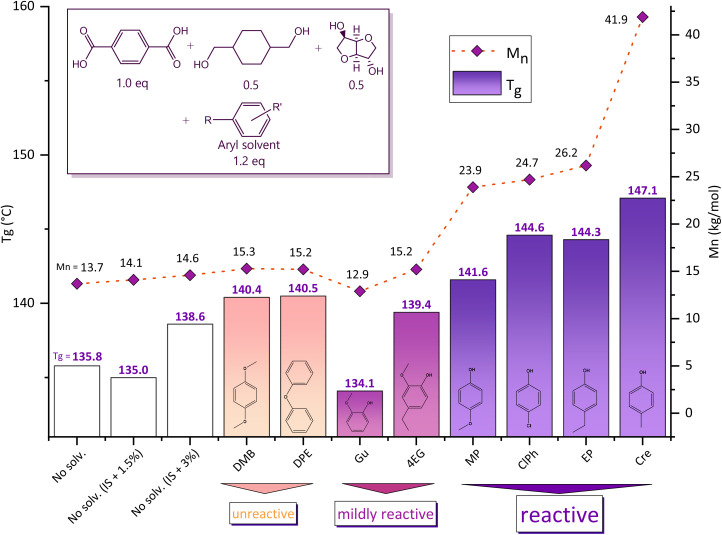
PICT results with the use of different solvents, reactive and unreactive, with 1.2 equivalent compared to the diacid. *M*_n_ is obtained by GPC and *T*_g_ by DSC (2^nd^ heating cycle). The dashed line for the molecular weights is there simply to help guide the eyes.

Our synthesis strategy makes use of phenolic reactive solvents for the production of polyesters. In Table 1 and Fig. 1, the synthesis of PICT is used to compare the reactive solvent method to conventional polycondensation routes and to the use of non-reactive solvents, with some comparative examples for PICF. In typical two-step melt polycondensation synthesis, feed ratios with excess of diol (diol : diacid >1) are used. The data in Table 1 shows that the PICT polyesters have a slight increase in molecular weight with increasing isosorbide excess in the feed (1.5, 3.0 and 10.0 mol%). The effect of diol excess can have different contributions: it can reduce the viscosity of the melt during (pre-)polycondensation and it can increase the diol end groups which can eventually react and give rise to chain growth. Especially for high viscosity melts, this is important because the high temperatures that are needed tend to cause isosorbide to leave the system (dragging). For the experiments shown in this work, all targeting a 50 : 50 diol ratio in the polymers, isosorbide was chosen as the diol to be in excess due to the higher reactivity of CHDM, with the objective of keeping the content of isosorbide in the final polymer constant. If too much CHDM were present in the system, it would tend to take over and be incorporated in excess (above 50%). Despite the improvements shown for PICT systems, typical two-step melt polycondensation methods do not seem capable of achieving higher molecular weights (*M*_n_ > 24 kg mol^−1^) for high isosorbide content polyesters in short reaction times (polycondensation ∼1 h).

Examples in the literature show that achieving high molecular weights in polyesters with isosorbide content above 30% is not an easy feat. Legrand *et al.*^10^ produced PICT polyesters and their efforts show how difficult it is to get past molecular weights of ∼16 kg mol^−1^ (*M*_n_). Semi-crystalline PICT polymers, below ∼35% isosorbide content, can be improved with solid state polymerization, but amorphous polyesters depend on a good synthesis pathway. Nonetheless, their results as well as ours (not presented in this paper) confirm that these molecular weights above 14 kg mol^−1^ are sufficient for ductile samples with good tensile and impact performance, as is the case of commercial materials like Tritan (PCTT).^22^ Other authors^9,23–26^ reported similar trends: it is possible to obtain PICT (and PEICT, in combination with ethylene glycol) with *M*_n_ ∼20 kg mol^−1^ at low isosorbide content, but polyesters with higher contents are almost inexistent. To the best of our knowledge, Yoon *et al.*^8^ obtained the best results with high isosorbide content for PCT-like polyesters, although their work included the incorporation of ethylene glycol into the polymer chains to improve reactivity. The use of reactive high boiling point phenolic solvents allows to circumvent these limitations.

The results shown in Table 1 and Fig. 1 indicate that the synthesis route with reactive solvents in PICT and PICF systems achieved low losses of isosorbide and high molecular weights (*M*_n_ > 24 kg mol^−1^) in the final polymers. It is especially remarkable that the synthesis with reactive solvents achieved such high molecular weights with only 1 hour of polycondensation of PICT, which performed much better than longer times for solventless reactions. This evidence suggests that the method with reactive solvents is capable of tackling the effective incorporation of unreactive monomers like secondary diols even with a 1 : 1 diol : diacid feed ratio (no excess of diol). Achieving virtually no loss of isosorbide with reactive solvents is easy at small scales, which can be verified by the high molecular weights obtained without diol excess (Table 1) and by the NMR quantification. The literature, on the other hand, shows that in typical methods of two-step melt polycondensation 10–30% of isosorbide is lost with a diol excess of 10–20%.^9,10^ The ability to minimize the loss of isosorbide is important to ensure the reproducibility of the syntheses and to manufacture materials with targeted composition. Combined with the improvement of polymer qualities, our synthesis method has low risk of errors and other difficulties like the addition of viscous components into an ongoing reaction system for compensation of diol/diacid proportions. To better understand the use of reactive phenolic solvents, we performed studies on the effect of different solvents on aromatic polyesters.

### Solvent effect

Different solvents tested for PICT systems are shown in Table 1 and Fig. 1 and range from unreactive to reactive. Unreactive solvents display some improvement in terms of *T*_g_ and *M*_n_ compared to typical solventless reactions, however they seem to reach a maximum *M*_n_ of ∼15 kg mol^−1^, similar to polymerizations with small excesses of diol. The best results are obtained with the reactive solvents: *p*-methylphenol (Cre), *p*-methoxyphenol (MP), *p*-ethylphenol (EP) and *p*-chlorophenol (ClPh). The amount of solvent end groups (EG) formed during esterification shows that these are the most reactive solvents on the list (for the ester-forming reactions), as opposed to mildly reactive ones, for instance. The amount of solvent end groups before polycondensation could be an indication that there is a threshold (minimum value) to allow sufficient chain growth during polycondensation, combined with other factors like leaving group ability. When forming no solvent end groups (unreactive solvent), or significantly smaller amounts as is the case with 4-ethyl guaiacol (4EG, end groups ∼8.7 mol%), the resulting polymer does not reach high molecular weight. This probably happens because, at the high temperatures required for isosorbide to react, the monomer tends to leave the system at a higher rate than the chain growth takes place. A higher amount of reactive leaving groups seems to overcome this barrier (*e.g.* Cre, end groups ∼19.6 mol%). A balance seems to be required for the method to occur: the solvent has to be reactive enough to form sufficient ester bonds with the diacid, but not too much as to hinder its leaving group ability. Additional effects like steric hindrance can also be relevant to the formation of the solvent end groups.

After more than 40 hours of (trans)esterification under nitrogen flow at reflux temperature, the reaction mixture with guaiacol was still inhomogeneous, showing that unreacted TPA was still present. The amount of Gu end groups formed (2.5%) after 40 h is very low, which shows how unfavourable Gu is for this reaction. This difficulty in converting the co-monomers is likely due to guaiacol's structure causing steric hindrance, which is a big obstacle to chain growth. Additionally, further difficulties arise from guaiacol's relatively low boiling point (205 °C) and the relatively low reactivity of terephthalic acid. In contrast, cresol has an even lower boiling point (202 °C), but higher overall reactivity, and therefore displays much better results and concludes (trans)esterification within 12–14 hours. This is an indication that the groups formed with reactive solvents like cresol are important to promote this reaction, which otherwise does not occur significantly at such low temperatures. Given the benefits provided by these solvents, we pursued a better understanding of their role in polyester synthesis.

Reactions performed in the presence of a reactive solvent have different causes for success:

1. *Activation and water removal*: due to its capacity to react with (di)acids, the solvent is capable of forming reactive esters with a better leaving group than water, the latter being removed from the system before PC. This enhances the reactivity of the (di)acid, promoting chain growth and easier interaction with less reactive co-monomers such as secondary diols (*e.g.* isosorbide). At the same time this prevents the formation of water during polycondensation, which is especially difficult to remove during the late stages. Since water gets replaced by the much less reactive aryl alcohols, the reverse reactions are reduced and higher molecular weights are possible. The activation and water removal seem to be the most significant factors.

2. *Reflux*: performing the reaction under reflux potentially hinders diols or diacids from escaping the reactor, since the solvent partially washes them down from the flask walls. It also heats up the reactor walls and part of the distillation path, facilitating the removal of water.

3. *Reduction of viscosity*: as the oligomers start to form and chains start to grow, the viscosity of the mixture/melt increases. This is especially relevant when incorporating high percentages of isosorbide (>30%), which increases the *T*_g_ significantly. Having a solvent present reduces the viscosity of the mixture, facilitating mass transfer (water removal) and allowing for lower esterification temperatures, in turn reducing loss of monomers by dragging or sublimation.

### Activation and water removal

In the first step of the synthesis, referred to as esterification, ester bonds between the diacid and the phenolic compounds are formed. This reaction increases the reactivity of the diacids by exchanging their leaving group, water, by a more active one such as *p*-methylphenol (cresol or “Cre”, p*K*_a_ = 10.14 ^27^ and bp = 202 °C). Eliminating water from the system during esterification (at low temperatures) prevents water from causing chain scission during polycondensation. The presence of a better leaving group now enables the diols to react more strongly with the activated acids, which is especially important for low-reactivity diols like isosorbide. Since isosorbide's p*K*_a_ is much higher (13.03)^28^ than the p*K*_a_ values of the reactive solvents (9.2–10.3), isosorbide is a worse leaving group and is, therefore, expected to remain in the chains while the solvent is eliminated as the reaction progresses. Among the solvents, cresol appears to display a good balance between a capacity to form esters with TPA and a good leaving group ability, which might explain its excellent performance. It is important to note that mass transfer plays a limiting role in high viscosity melts,^29^ therefore this factor must always be taken into account when understanding reactivity. Similar substituted phenols were tested: *p*-ethylphenol (“EP”, p*K*_a_ = 10.00 ^30^ and bp = 218 °C) and *p*-methoxyphenol (“MP”, p*K*_a_ = 10.21 ^27^ and bp = 243 °C). These solvents displayed good results as well, though their higher boiling points might be a reason for their worse performance, due to the longer times required to distil out such solvents and their end groups. Another practical consideration is that EP and MP may solidify at room temperature, which can cause clogging in distillation processes if care is not taken. Following the end group formation during esterification may further clarify these dynamics and differences in performance.

**Fig. 2:**
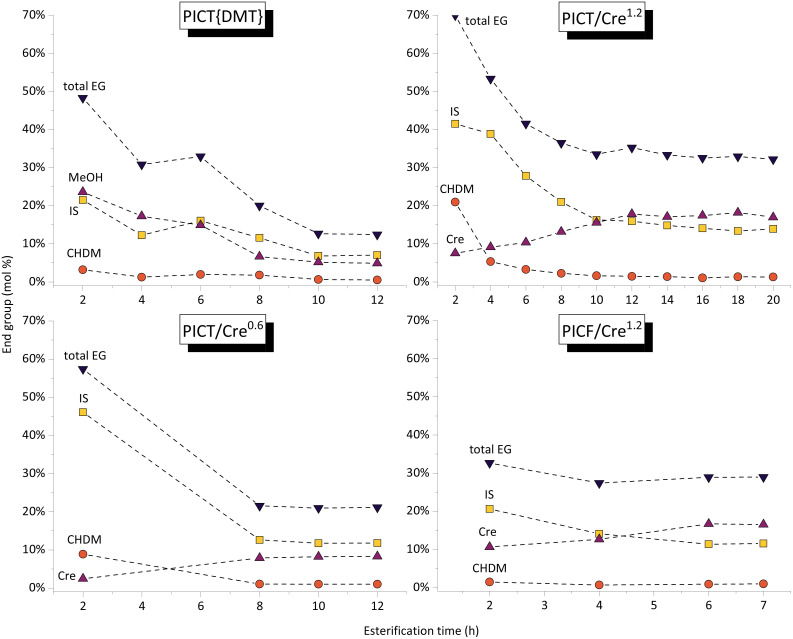
End group evolution in different batches of PICT and PICF, using Cre as solvent, calculated from ^1^H-NMR spectra. The data points presented here are measured during esterification only (under nitrogen flow, at ambient pressure). “PICT{DMT}” shown here (without solvent) uses dimethyl terephthalate, DMT, as precursor, not TPA as the other reactions. The superscript shows the amount of solvent used, in eq., compared to the diacid. Total end group (EG) estimated without titration – acid EGs were not accounted for. Dashed lines are for visual assistance only.

The formation of end groups of reactive solvent and diols, followed by ^1^H-NMR, tells us about the chain growth of oligomers. The end group quantification method is provided in the ESI.†Fig. 2 shows how, during (trans)esterification, the increase of molecular weight reaches an equilibrium state, observed by the plateaus. This is a good indication that at this point the system requires the polycondensation step – higher temperature and vacuum – for chain growth. There are significant differences to be noticed among the presented systems in terms of reaction time and chain length of oligomers.

The rate of esterification with reactive solvents is strongly dependent on their reactivity and their boiling points, assuming no overpressure is used. The PICT system with cresol (1.2 eq., bp = 202 °C) takes about 14 hours to reach equilibrium at reflux temperature, while the system without solvent or with a higher boiling point solvent like MP (1.2 eq., bp = 243 °C) reaches equilibrium in about 8 hours. Although the solvents display differences in reactivity, it is likely that the reaction temperature is more significant in reducing reaction time in this comparison. Replacing TPA for FDCA tends to accelerate the (trans)esterification due to FDCA's higher acidity – around 6 hours are sufficient for PICF with the same amount of cresol as PICT. Besides the difference in reaction times, chain growth during esterification depends on solvent type and amount as well.

The amounts of end groups (EG) in equilibrium could indicate that the oligomers formed with higher amounts of solvent tend to be shorter (more total end groups, lower *M*_n_) in comparison to solventless systems or reactions with less solvent (ESI, Table S1†). Titration of acid end groups could be conducted to get more accurate numbers for end group composition – the molar amounts of acid end groups are not taken into account in the current calculations. In all reactions in Fig. 2, isosorbide EGs show to be the least reactive among diol EGs: they take longer to reach an equilibrium and they remain as the predominant diol EG.

In the first case shown in Fig. 2, DMT was used as a precursor and as such all acid end groups are capped either by diols, solvents, or methanol, which enables better quantification than the other cases. It is important to note that using DMT as monomer implies that only transesterification reactions are taking place, while reactions with TPA will undergo esterification followed by transesterification reactions (when alcohol or solvent end groups are present). With these considerations, by evaluating the total amount of EG in equilibrium, considerably fewer end groups form without the use of solvent (∼12%) when compared to systems with MP or Cre (∼30–40%). This contrast could indicate that the solvent molecules keep breaking down the oligomer chains as they form, preventing longer chains during esterification. Since the boiling points of the solvents are high and we maintained them under reflux, the solvents cannot leave the system as water or methanol would, which would in turn promote chain growth and prevent the backward reaction. Despite not growing as much, the chains activated by Cre or MP end groups show to be much more reactive and (virtually) no more water is formed after equilibrium is reached. Polycondensation, therefore, occurs more rapidly and to a greater extent, as shown in Fig. 1. In this context, there likely is an optimal amount of solvent in the feed, which allows the formation of enough active end groups and therefore produce high molecular weight polyesters.

**Fig. 3:**
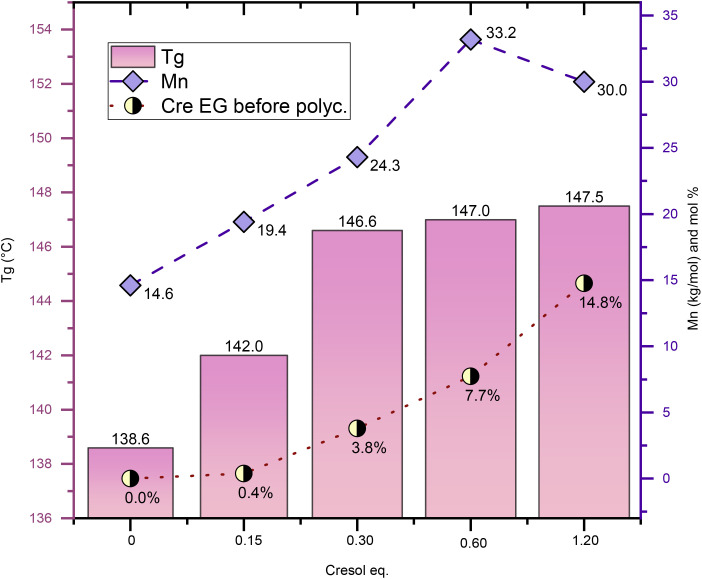
Solvent optimization for PICT using cresol, all batches with 3% of isosorbide in excess in feed (diol/diacid = 1.03). The equivalent of cresol used is in relation to the equivalent of terephthalic acid used in the feed. All solvent-assisted reactions shown underwent 2 hours of polycondensation. More data from these experiments in the ESI.†

Fig. 3 shows experiments aimed at optimizing the amount of solvent for PICT systems, in which different molar quantities of Cre were tested, ranging from 0 to 1.2 equivalent (relative to TPA). These experiments use 3% diol (IS) excess in the feed to compensate for potential loss of isosorbide. This is especially relevant for reactions with very small amounts of solvent. Likely as a result of this excess, which caused a deviation from the 1 : 1 diol : diacid ratio, the final polymer PICT/Cre^1.2^_(IS+3%)_ has lower *M*_n_ compared to the one with no IS excess (PICT/Cre^1.2^) shown in Table 1. The results indicate that, above 0.15 eq. solvent, there is evident improvement in the quality of the formed polymers. PICT/Cre^0.15^_(IS+3%)_ required higher oil temperature (∼265 °C) to be stirred due to an insufficient reduction of the system's viscosity. Overall, if too little solvent is used, an insufficient amount of solvent EGs is likely formed, leading to low molecular weights. At higher amounts of solvent, such as 0.6 and 1.2 eq., the ratio of end groups of solvent/diol at the end of esterification approaches 1, which likely facilitates chain growth (Cre EG/diol EG ∼0.65 for 0.6 eq. and 0.91 for 1.2 eq. – see Table S2†). This happens because, as polycondensation progresses, virtually each isosorbide EG can find one Cre EG to react with, thus allowing for high molecular weight polymers.

For some of the other solvents, however, the number of solvent end groups is not enough for improving the polyester. Such is true for PICT/4EG^1.2^, which formed more end groups (8.7%) than PICT/Cre^0.6^_(IS+3%)_ (7.7%), yet performed much worse. This could relate to 4EG's worse leaving group ability and to its reactivity. For suitable solvents like MP and Cre, there seems to exist an optimal range for solvent amount. Taking into account that less solvent is desired to avoid costs associated with purification, distillation and usage of reactor space, and that the molecular weight does not significantly change between 0.6 and 1.2 eq., this range seems adequate for PICT polyester production with *p*-cresol as solvent.

It is relevant to note that for cases of high *M*_n_ (high viscosity), effects of mass transfer, ineffective stirring and difficulty in sampling may influence the accuracy and reproducibility of the results. The amount of solvent, therefore, must be evaluated for each type of reactor in order to form enough reactive EGs, to have sufficient viscosity reduction and to account for mass transfer differences, thus producing high quality polymers. Similarly, polycondensation times have to be evaluated depending on the expected MW for each application.

**Fig. 4:**
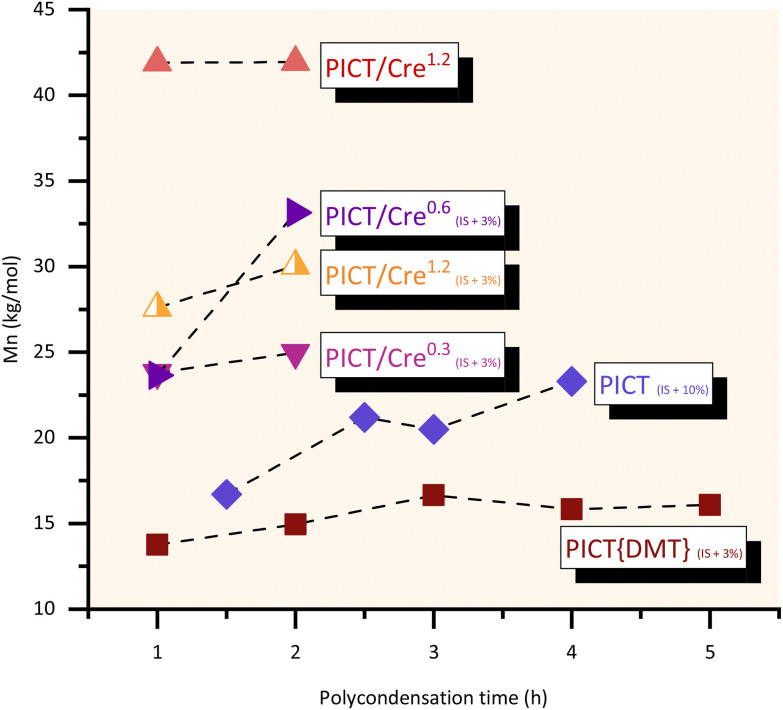
Influence of polycondensation times for different systems of PICT. The subscript represents the diol (IS) excess present in the feed, if any, while the superscript shows the equivalents of solvent used when relevant. *T*_PC_ = 285 °C, *t*_pc_ = 10–17 h (until equilibrium of end groups). More data in the ESI.†

Different polycondensation times were evaluated for the polyesters in this study and are shown in Fig. 4, including a reaction with a relatively high excess of 10 mol% of isosorbide in the feed (1.1 diol : diacid ratio). This higher excess served to compare the use of a (significant) diol excess with the use of a high performance reactive solvent. Relatively short polycondensation times of 1–2 hours are enough to provide high molecular weight PICT polyesters when promoting the synthesis with reactive solvents. Reactions with cresol seem to be better without the use of excess of diol, reaching a high plateau in *M*_n_ even in one hour of polycondensation. For cases without solvent at small scale, increasing isosorbide excess to levels around 10% and polycondensation times up to 4 hours can then give significant improvement in molecular weights (*M*_n_ ∼23 kg mol^−1^). The choice of synthesis method, therefore, must take into account what molecular weights are necessary for the desired polymer application, and if it is worth investing in longer polycondensation (vacuum) time, which is expensive, when not using solvents.

### Reflux and reduction of viscosity

In the absence of solvents, high esterification temperatures are required: to react the lowly reactive isosorbide and to reduce the system's viscosity. After 2–3 hours of esterification, the PICT system forms enough oligomers to shift the mixture's melting range to above ∼240 °C. Using (oil) temperatures around 250–260 °C, however, will cause considerable amount of isosorbide and terephthalic acid to leave the system through dragging and/or sublimation, which offsets the ratio of diol to diacid. The prevention of monomer loss has been easily evidenced by comparing systems with and without solvent under the same parameters. An example for PICT without solvent in the ESI (Fig. S7†) shows significant deposition of isosorbide onto the distillation path while under nitrogen flow. Using a solvent reduces the system's viscosity, which in turn may reduce loss of monomers and facilitate the removal of water. High boiling solvents (>200 °C) enable the system to be kept at a temperature high enough to react isosorbide with terephthalic acid, but low enough to keep these monomers from leaving the reaction system.

During the formation of oligomers under nitrogen gas flow, the temperature is selected such that the solvent is at reflux. This enables the solvent to heat up the reactor and condensation path, which potentially facilitates the removal of water. The reflux may partially wash down the isosorbide and other components that might have been removed from the mixture. A reflux at too high temperature, however, will not avoid loss of isosorbide – such as evidenced by the reaction with the unreactive solvent DPE (bp = 258 °C).

### Solvent elimination

**Fig. 5:**
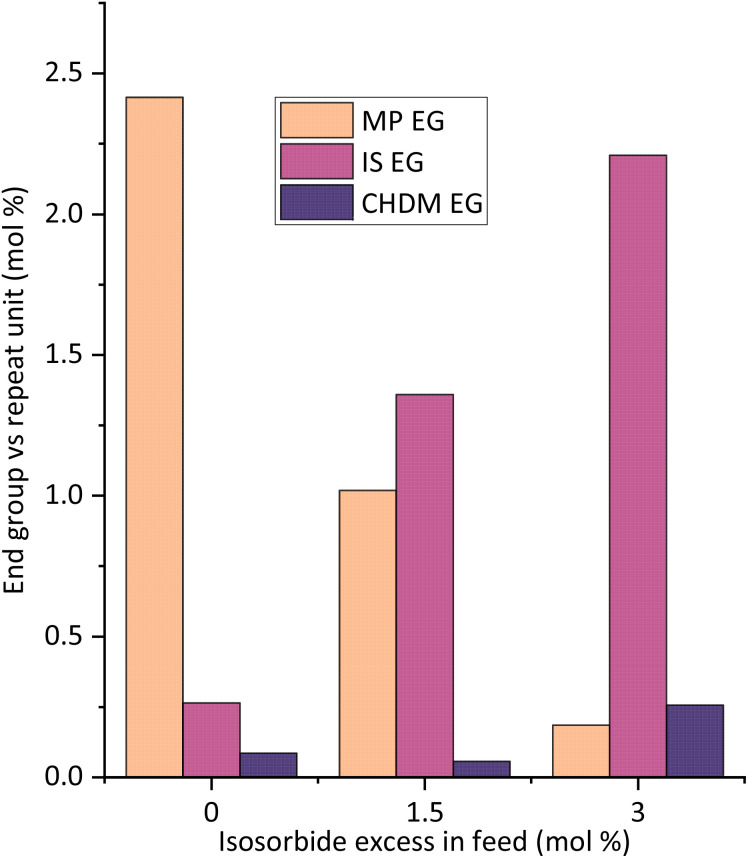
End groups in mol percent *versus* the repeat unit according to different reactions with different IS excess in feed for PICT/MP^1.5^ (1.5 eq. of MP solvent). The three polymers are in a narrow range of molecular weights (*M*_n_ = 18.9–19.3 kg mol^−1^) and *T*_g_ (141.7–142.5 °C).

The esters formed between reactive solvent and diacid are important for chain growth, yet they might remain as end groups in the polymer. For the aryl solvents studied, the solvent end groups are typically visible on the ^1^H-NMR spectra, as shown on Fig. 5. For solvent amounts ∼0.6 eq., mostly diol end groups are present in the chains at the end of esterification (Fig. 2). For large amounts of solvents (*e.g.* 1.0 MP eq.), however, the aryl esters tend to be present in quantities similar to those of diol end groups. For such cases, since isosorbide EGs are considerably unreactive, especially the endo hydroxyl groups, these chains possibly reach a virtual “dead end” with mostly solvent EGs remaining. As a consequence, the polymer product could have these phenolic solvent EGs present, which are undesirable.

**Fig. 6:**
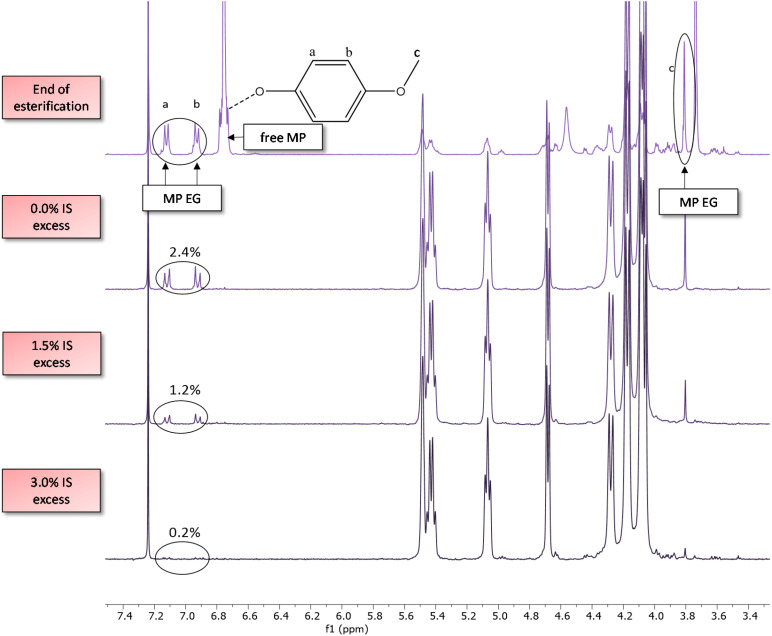
Elimination of solvent end groups by use of a small excess of isosorbide (in feed) for PICT/MP^1.5^ (1.5 eq. of MP solvent). The spectrum at the top shows a sample at the end of esterification, while the other ones show polymer samples (end of reaction). The NMR spectra show the end groups of 4-methoxyphenol (MP) ester with terephthalate units, both in the aromatic region ∼7 ppm and in the methoxy region ∼3.75 ppm.

The complete removal of solvent (end groups) is desired for safety reasons (toxicity) and to avoid potential solvent smell, especially for more sensitive applications like food contact. In an attempt to eliminate solvent EGs, a small excess of isosorbide was added in the feed of different reactions (Fig. 5 and 6). With this excess, the amount of solvent EGs was significantly reduced. As little as 3.0 mol% excess of isosorbide (feed), relative to the repeat unit, is virtually enough to eliminate MP end groups from the chain, from 2.4% solvent EG (no excess) to ∼0.2% on the shown example. Longer polycondensation time could reduce the solvent EGs even further. With the diol excess, isosorbide now becomes the major end group, going from 0.3 mol% (no excess) to 2.2 mol% (3% IS excess). Upon substitution of the aryl EGs, small amounts of solvent will be liberated and subsequently removed from the melt, and for that process the boiling point of the solvent is relevant.

A reactive solvent's boiling point, for our method, should be low enough to facilitate its removal from the system at the end of polycondensation. On the other hand, if the boiling point is too low and the solvent is not reactive enough, such as is the case with guaiacol (205 °C), the reaction may take an unfeasibly long time. For these reasons, solvents that fit into the balance of reactivity *vs*. boiling point like cresol are preferred, or else solvents with slightly lower boiling points may be considered in combination with overpressure. Additionally, the surface area for distillation in the reactor must be taken into account, which may be an obstacle depending on the scale used. Designs of reactors which present high surface areas for distillation, such as disk reactors, may facilitate the elimination of all traces of solvent and the fast achievement of high molecular weights at large scales.

### PICT from dimethyl terephthalate (DMT)

The main focus in this paper is to study reactions that use terephthalic acid (TPA) as starting diacid source; nonetheless, dimethyl terephthalate, DMT, is an important alternative monomer to be considered in polyester synthesis. The commercial production of DMT from fossil resources is much lower (∼1 Mtpa)^31^ than that of TPA (>70 Mtpa).^32^ Considering renewable sources, however, DMT is an important candidate, as it can be obtained from chemical recycling of poly(ethylene terephthalate), PET, *via* methanolysis.^6^ For polymer synthesis, DMT is especially useful in reactions in which acidity is a problem. This is the case, for instance, for systems with 1,4-butanediol, which easily undergo acid catalysed dehydration in presence of (di)acids like TPA, forming tetrahydrofuran (THF). With these aspects in mind, we researched the possibility of producing polymers from DMT using the present methodology with reactive solvents.

The reactions with dimethyl terephthalate, in Table 2, show some ease in handling compared to TPA. DMT melts around 140 °C, therefore the whole mixture is homogeneous from the start of the reaction, which allows for better quality of NMR samples, since they can be completely dissolved in deuterated chloroform (no free TPA present). Additionally, there might be less loss of co-monomers compared to TPA reactions, which can be observed from the cleanliness of the glass reactor. The homogeneity of this reaction system might facilitate mass transfer, allowing for incorporation of isosorbide – thus increasing molecular weight – before the melt needs higher temperatures, above 240 °C. Additionally, methanol has a lower boiling point than water, which could make its removal easier from (viscous) polymer melts.

**Table 2:** Comparison between PICT polyesters synthesized from dimethyl terephthalate, {DMT}, and from terephthalic acid, TPA. All displayed samples have 3 mol% excess of isosorbide in the feed (subscript). The other synthesis conditions were equivalent to the TPA-based reactions: esterification temperature around 240 °C, increased to 285 °C at the start of polycondensation (full vacuum). The exception was PICT_(IS+3%)_, which needed higher temperature (260–280 °C) during esterification due to the absence of solvent

Sample	Precursor/solvent [solv. equiv.]	Composition in polymer IS/CHDM (mol%)	*M*_n_ [*Đ*] (kg mol^−1^)	*T*_g_ (°C)	*t*_est._/*t*_polyc._ (h)	Solvent EG before vacuum (mol%)
PICT_(IS+3%)_	TPA/no solv.	49.6/49.8	14.6 [2.5]	138.6	9.3/3.3	0.0
PICT/Cre^1.2^_(IS+3%)_	TPA/Cre [1.2]	50.3/50.2	30.0 [2.1]	147.5	16.7/2.0	16.0
PICT{DMT}_(IS+3%)_	DMT/no solv.	48.5/49.9	16.6 [3.2]	137.8	14.0/3.0	0.0
PICT{DMT}/Cre^1.1^_(IS+3%)_	DMT/Cre [1.1]	51.1/48.9	24.5 [2.3]	142.9	17.0/3.0	13.5

Overall, the use of DMT as precursor (PICT{DMT}_(IS+3%)_) shows a slight improvement compared to TPA for solventless reactions, but worse results with the reactive solvent cresol. Starting from DMT caused the dispersity (*Đ*) to increase significantly from the usual values of 2.0–2.5 to 3.2. From the data obtained so far it is unclear to us why *Đ* is so much higher for PICT{DMT}. When analysing the cases with cresol, the transesterification reaction between DMT and cresol produced a comparable content of solvent EGs (13.5 mol%) in comparison to the reaction using TPA (14.8 mol%). It would therefore be expected that the polyester from DMT would reach a molecular weight close to 30 kg mol^−1^ (*M*_n_), taking into account the data from Fig. 3. This was, however, not the case: the *M*_n_ achieved by PICT{DMT}/Cre^1.1^_(IS+3%)_ was 24.5 kg mol^−1^, even with one extra hour of polycondensation time compared to the TPA case PICT/Cre^1.2^_(IS+3%)_. The main factor is likely the presence of methanol EGs (∼5 mol%) in the esterification equilibrium stage. The MeOH EGs undergo a different mechanism than the acid end groups of TPA, which may have hindered chain growth. Overall, it seems reasonable that PICT can be obtained from DMT with high molecular weight with the use of reactive solvents, but more studies should be performed to understand and optimize this system.

### Scale-up

**Fig. 7:**
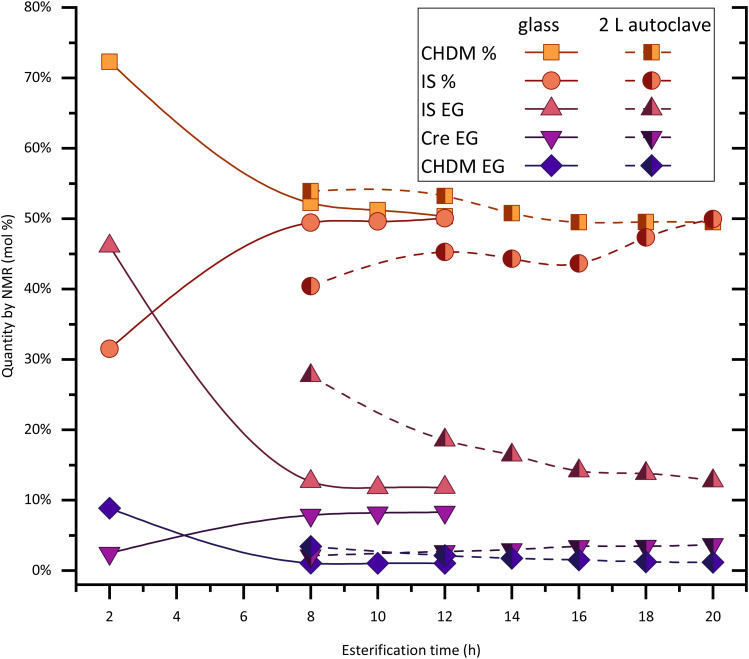
Scale-up (trans)esterification reaction for PICT in 2-liter autoclave (half-painted symbols) with 0.3 equivalent of cresol compared with small-scale reaction in 100 ml glass reactor (filled symbols) with 0.6 equivalent of cresol. No data on this graph regarding the vacuum steps. Lines/dashed lines are only meant to help with visualization.

Experiments of PICT in larger scale (∼850 g of polymer) were performed with different solvents and conditions (Fig. 7 and Table 3). A larger scale like this provides advantages like sturdier stirring, which likely translates to higher homogeneity. There are, however, difficulties with mass transfer that should be considered. The esterification reaction in our autoclave system takes considerably longer than at 20 g scale, due to the removal of condensation products (water, solvent) made difficult by the reactor's smaller distillation area per volume ratio. Our results show that without overpressure, the reaction of PICT in an autoclave with 0.3 eq. of cresol can take around 20 hours to reach an equilibrium, while a reaction in 20 g scale with 0.6 eq. of cresol, which should be slower (more of lower boiling solvent), reaches equilibrium in 8–10 h (see Fig. 2). Additionally, other effects can take place in the autoclave, like the larger temperature gradient leading to hotter zones than in the glass, and the potential coagulation of terephthalic acid particles, which cannot be visually confirmed in the autoclave but can be identified in glass reactors. Previous results by our group, however, show that the kinetics can be less hindered in a large system, with this method, if the viscosity of the melt is lower and the reactivity is higher. Such is the case of the esterification of PISA (succinic acid and isosorbide) using cresol, which takes around 7 hours in the 2-liter autoclave and 5 hours at 20 g scale.^21^ The high viscosity brings another challenge: the extrusion of the polymer from the reactor.

**Table 3:** PICT scale-up: synthesis in 2-liter autoclave (750–920 g per batch). Subscript represents isosorbide excess in feed, if any

Sample	*M*_n_ [*Đ*] (kg mol^−1^)	*T*_g_ (°C)	Composition in polymer IS/CHDM (mol%)	Overpressure (bar)	*t*_est._/*t*_polyc._ (h)	Excess IS in feed (mol%)	*T*_esterif._/*T*_PC_ (°C)
PICT/Cre^0.3^_(IS+3%)_	18.2 [2.4]	141.2	50.1/50.0	0 (ambient P)	20/2.5	3	240/285
PICT/EP^1.0^_(IS+3%)_	20.0 [2.0]	139.7	49.9/49.5	0	23/1.2	3	200–260/280
PICT/EP^1.2^	16.4 [2.1]	138.0	48.9/50.7	2.2–2.7	8/2.5	0	240–280/280
PICT/MP^1.0^_(IS+3%)_	15.1 [2.1]	135.0	48.2/48.8	0	6/4.3	3	260/285
PICT/Gu^1.5^	10.9 [2.8]	135.4	51.9/47.4	2.7–3.2	8/3	5	240–290/285

The molecular weight limit for PICT that seems extrudable in our autoclave system is around 16 kg mol^−1^ (*M*_n_). Higher molecular weights (18–20 kg mol^−1^) take long hours at high temperature and therefore may degrade the polymer back to ∼14–15 kg mol^−1^, as witnessed for PICT/Cre^0.3^ and PICT/EP^1.0^ (Table 3). These *M*_n_ values must be understood in combination with the presence of the ring-opened form of isosorbide (through ring hydration), which can cause chain branching and therefore increase the viscosity of the polymer. This compound could sometimes be identified in our polymers by the presence of insoluble small particles in DCM or CDCl_3_, and through rheology analysis. Quantifying and avoiding the formation of this compound are important steps for improving the extrusion of isosorbide-containing polyesters at large scales. Additionally, the synthesis can be stopped before reaching higher molecular weights, since tensile and impact bars of PICT at *M*_n_ > 14–15 kg mol^−1^ present ductility and good overall performance (not shown on this paper). Finally, better extrusion systems can be considered for such high *T*_g_ polymers, like the use of larger nozzles, different reactor designs, extrusion pumps *etc*.

Results on Table 3 show that cresol and EP perform very well for the synthesis of PICT, in agreement with small scale experiments. Due to mass transfer limitations inherent to this system, however, the achieved molecular weights are significantly lower than those at smaller scale. Cresol, even in amounts as small as 0.3 eq., can provide high molecular weights for PICT. EP performs very well too, though systems with these two solvents take a long time (low boiling points) to achieve equilibrium in esterification. PICT/EP^1.2^ shows that esterification time can be significantly shortened by the use of overpressure and high molecular weights can still be achieved in that way. MP, on the other hand, displayed a tendency to form an ether compound with CHDM that was probably preventing further chain growth (chain capping – ESI†). For this issue, ether suppressants like sodium phosphate can be added, and milder temperature profiles may be considered. Guaiacol, as also shown at smaller scale, performs worse than the other solvents due to its hindered structure. The results seem to provide evidence that reactive solvents like cresol and EP can play a decisive role in producing aromatic polyesters with high isosorbide content at large scales with sufficiently high molecular weight. As a further advantage towards scalability, the reactive solvents like *p*-cresol can be recycled multiple times without significant loss in performance, as also evidenced by Weinland *et al.*^21^

## Conclusions

The results of this study indicate that the use of high-boiling point reactive aryl solvents can facilitate the synthesis of certain polyesters. This method may be especially useful when overcoming challenges like the incorporation of a secondary diol such as isosorbide, whose reactivity is lower than (typical) primary diols. This synthesis route works especially well in promoting the formation of aromatic and aliphatic polyesters with high molecular weights and high incorporation of isosorbide. It is important to note, however, that thermally stable polymers like PICT might reach sufficient molecular weights if enough diol excess is applied as shown in our results. With these two cases in mind – solvent *vs.* excess diol – one must choose if the use of a solvent is justified for its advantages – reducing polycondensation time for example, or if an excess of diol is preferred, although longer polycondensation times are needed to achieve high MW. These considerations are especially important for large scale operations, in which high MW's can lead to difficulties in extrusion for highly viscous materials like PICT, while lower molecular weights might suffice (∼15 kg mol^−1^) for most applications.

Further investigation should be conducted aiming to optimize the removal of the residual reactive aryl solvent (end groups) from the polymer, given their toxicity, but the results presented in this paper clearly indicate the high potential of the solvent elimination strategy. Additionally, further knowledge about eliminating the ring-opened compound of isosorbide is important for further scale-up.

## Author contributions

Conceptualization: G. J. M. G., B. W., D. H. W. and B. B. P.; writing: B. B. P., G. J. M. G. and R. J. P.; experimentation: B. B. P., N. G. and S. V.; characterization and analyses: B. B. P.

## Conflicts of interest

There are no conflicts to declare.

## Supplementary Material

PY-014-D2PY01578A-s001
