# Supplementary material for: Reactive phenolic solvents applied to the synthesis of renewable aromatic polyesters with high isosorbide content
Source: Polym Chem. 2023 Jun 26;14(27):3225–38. doi: 10.1039/d2py01578a (PMC10334470; doi:10.1039/d2py01578a)
Supplement: PY-014-D2PY01578A-s001 [file PY-014-D2PY01578A-s001.pdf]

Electronic Supplementary Material (ESI) for Polymer Chemistry.
This journal is © The Royal Society of Chemistry 2023

Supplementary Information

Reactive Phenolic Solvents Applied to the Synthesis of Renewable Aromatic Polyesters with High Isosorbide Content

Bruno Bottega Pergher[a], Narcisa Girigan[a], Sietse Vlasblom[a], Daniel Weinland[a], Bing Wang[b], Robert-Jan van Putten[a, b] and Gert-Jan M. Gruter* [a, b]

[a.] *Van't Hoff Institute of Molecular Sciences, University of Amsterdam, P.O. Box 94720, 1090GS Amsterdam, The Netherlands.*
[b.] *Avantium Chemicals BV, Zekeringstraat 29, 1014BV Amsterdam, The Netherlands.*

* Corresponding author: Gert-Jan Gruter, g.j.m.gruter@uva.nl.

.

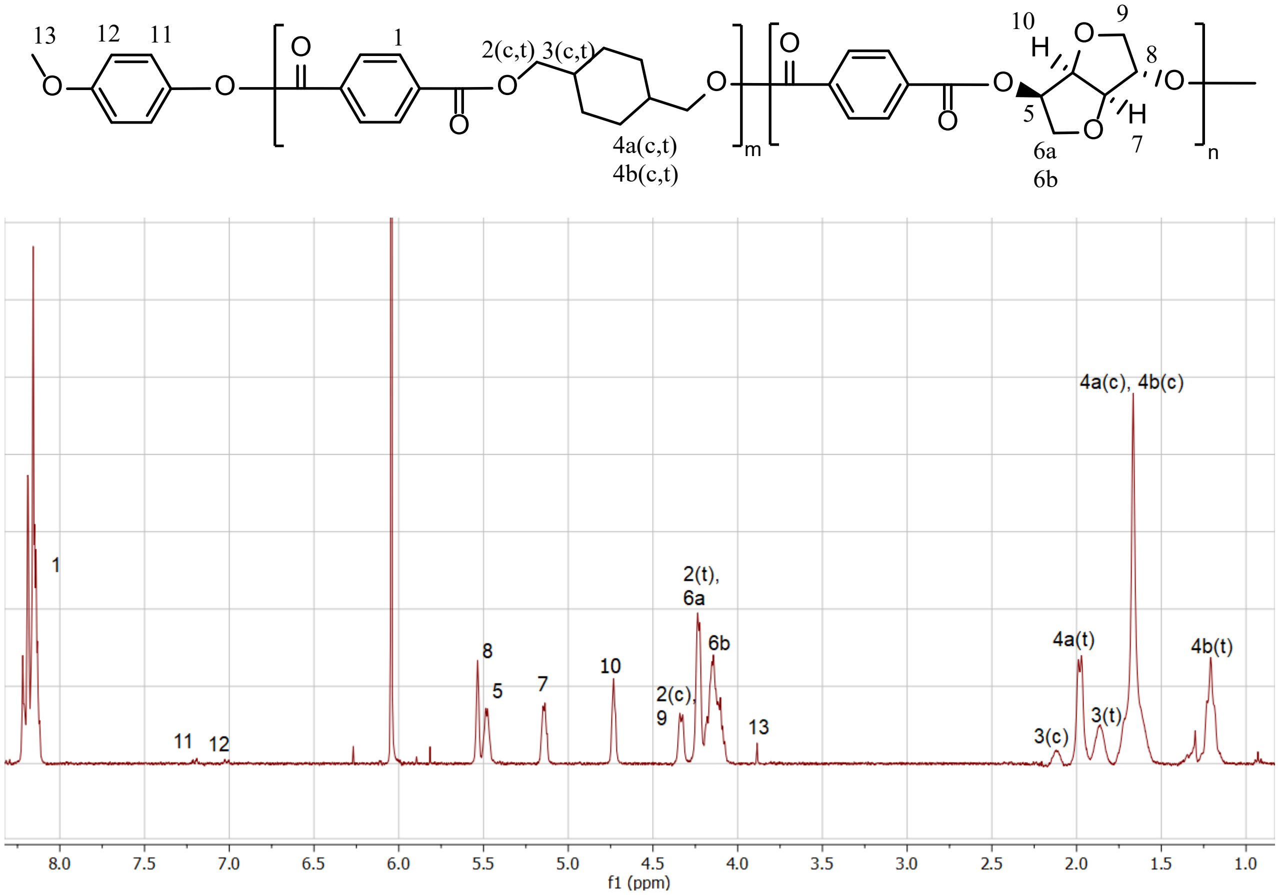

Figure S1 ^{1}H-NMR and structure of PICT with p-methoxyphenol (MP) end groups present and respective peak assignment.

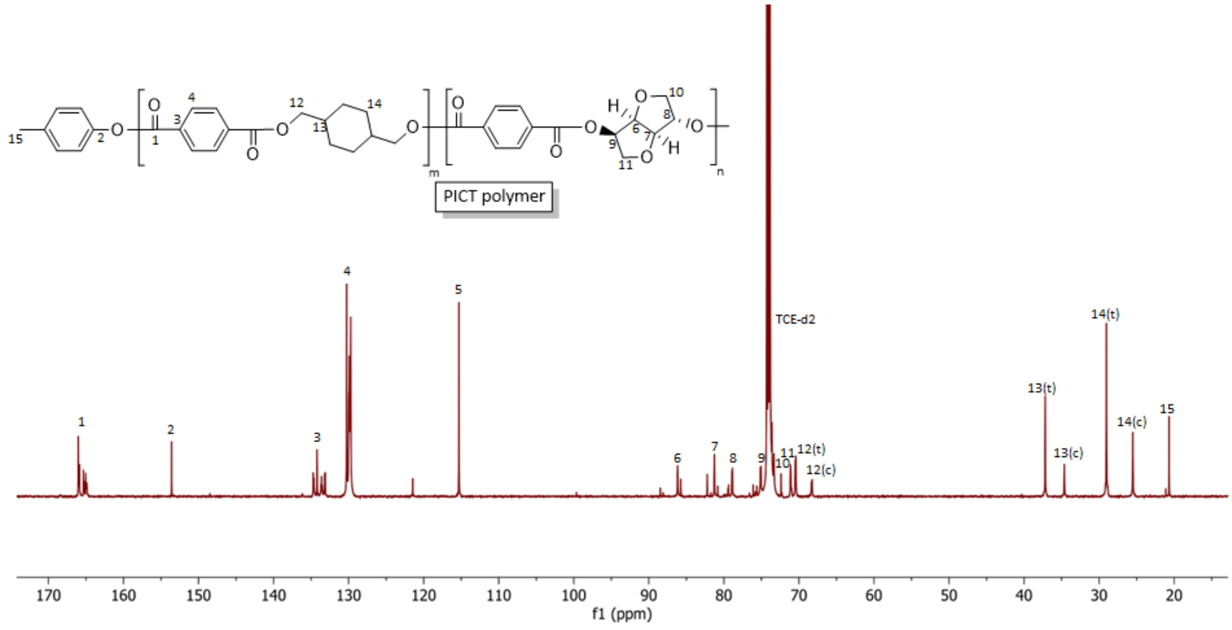

Figure S2 ^{13}C-NMR of PICT/Cre[1.2] polymer before vacuum step. Spectrum read with sample dissolved in TCE at 18h of a reaction performed in a 2 L autoclave, using 0.3 equivalent of cresol compared to terephthalic acid.

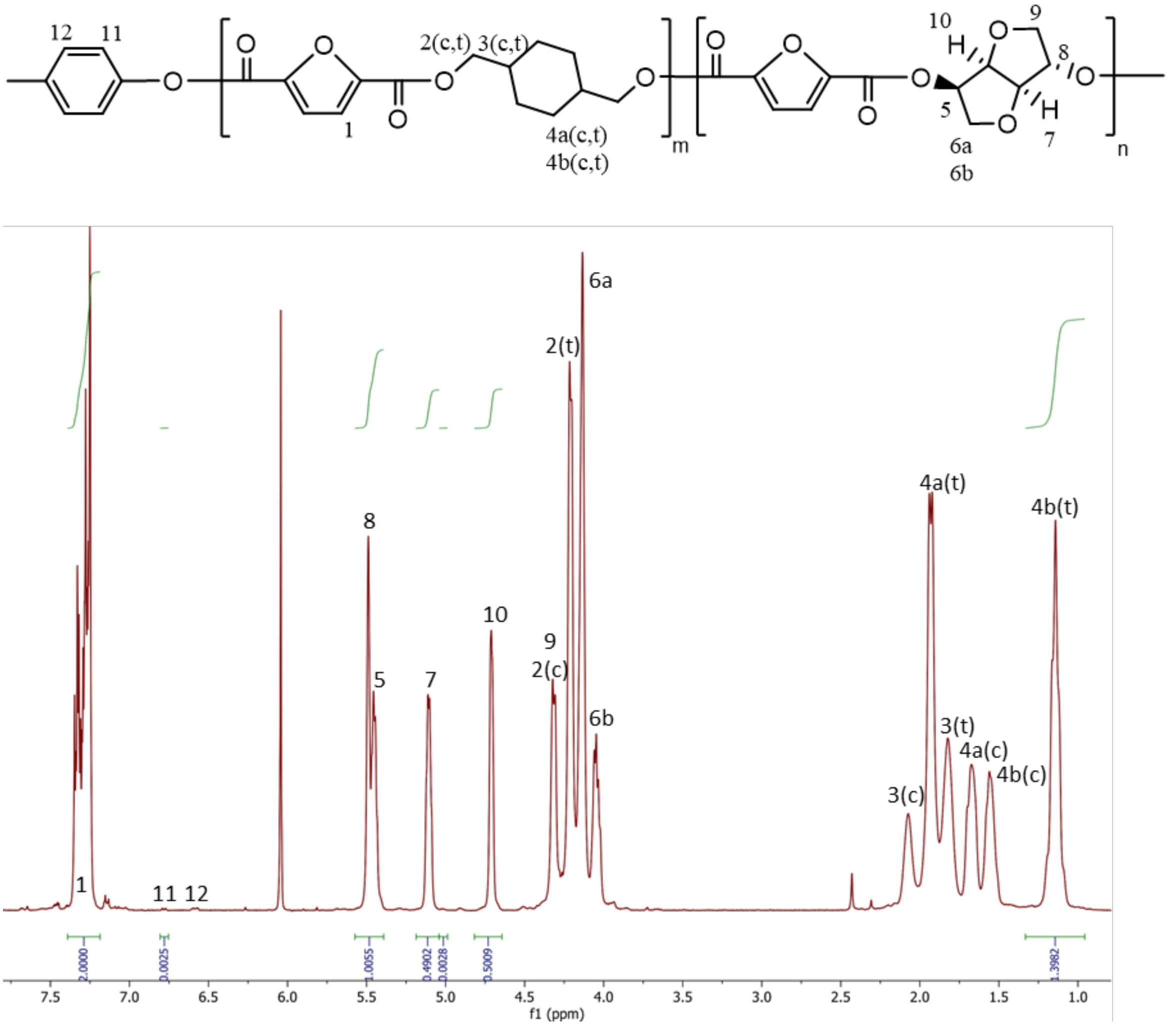

Figure S3 PICF proton NMR. “c” and “t” refer to cis and trans, respectively.

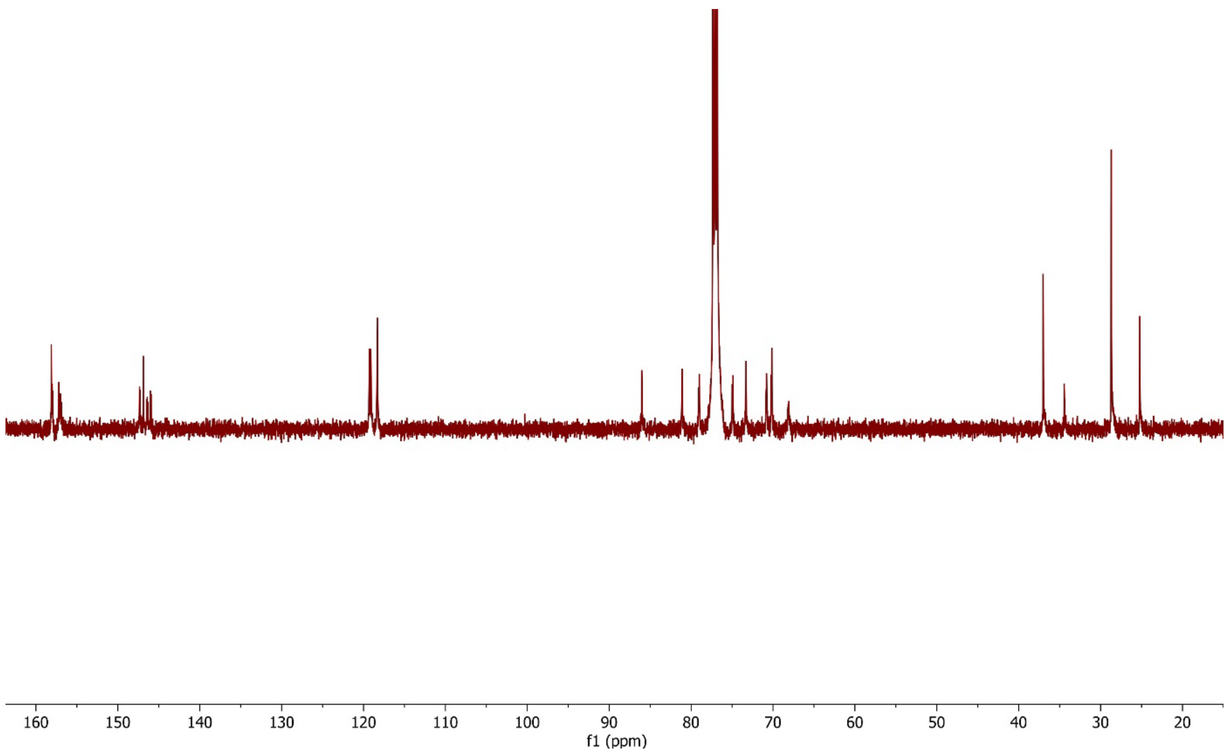

Figure S4 ^{13}C-NMR of PICF/Cre$^{1.2}$ polymer sample. Most peaks are the same as PICT/Cre$^{0.3}$ (previous image), except for the furanoate peaks and the absence of p-cresol peaks – this last difference due to being a polymer sample with virtually no p-cresol present.

Figure S5 shows a PIT reaction (no CHDM), with peak assignments used for calculation of end groups. The endo groups are predominant, in accordance with the literature.[1–3] The assignment of end groups for CHDM, shown on Figure S6, is in accordance with Amari et al.[4]

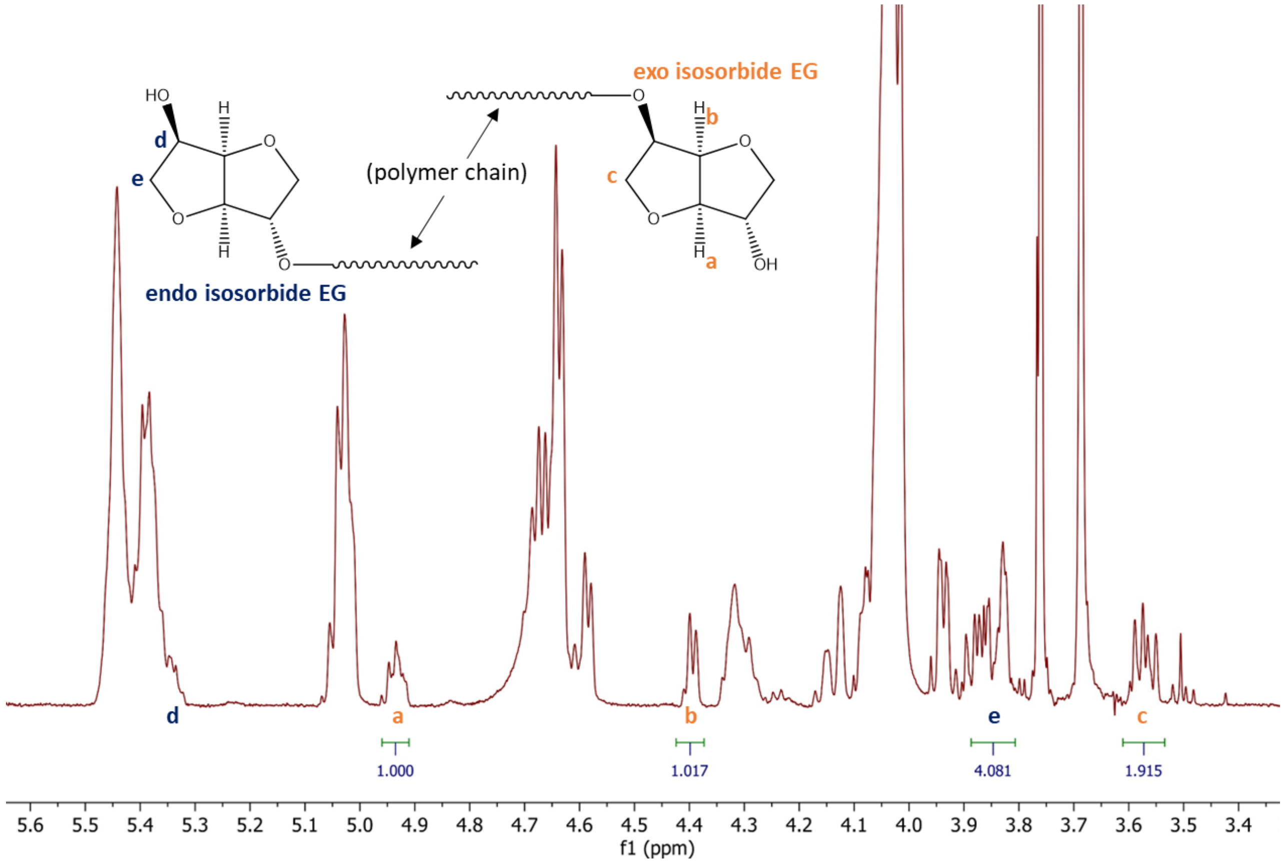

Figure S5 Assignment of end groups of isosorbide from a PIT (poly(isosorbide terephthalate)) sample, in accordance with Yoon et al. The reactivity difference between endo and exo end groups are very similar to reported in the literature. This can be verified by the integral proportions between endo and exo, which is close to 1:2 (normalized to 1 proton). Endo groups, for being more hindered, represent the majority of end groups present in PIT and PICT, around 2/3 of the total isosorbide end groups.

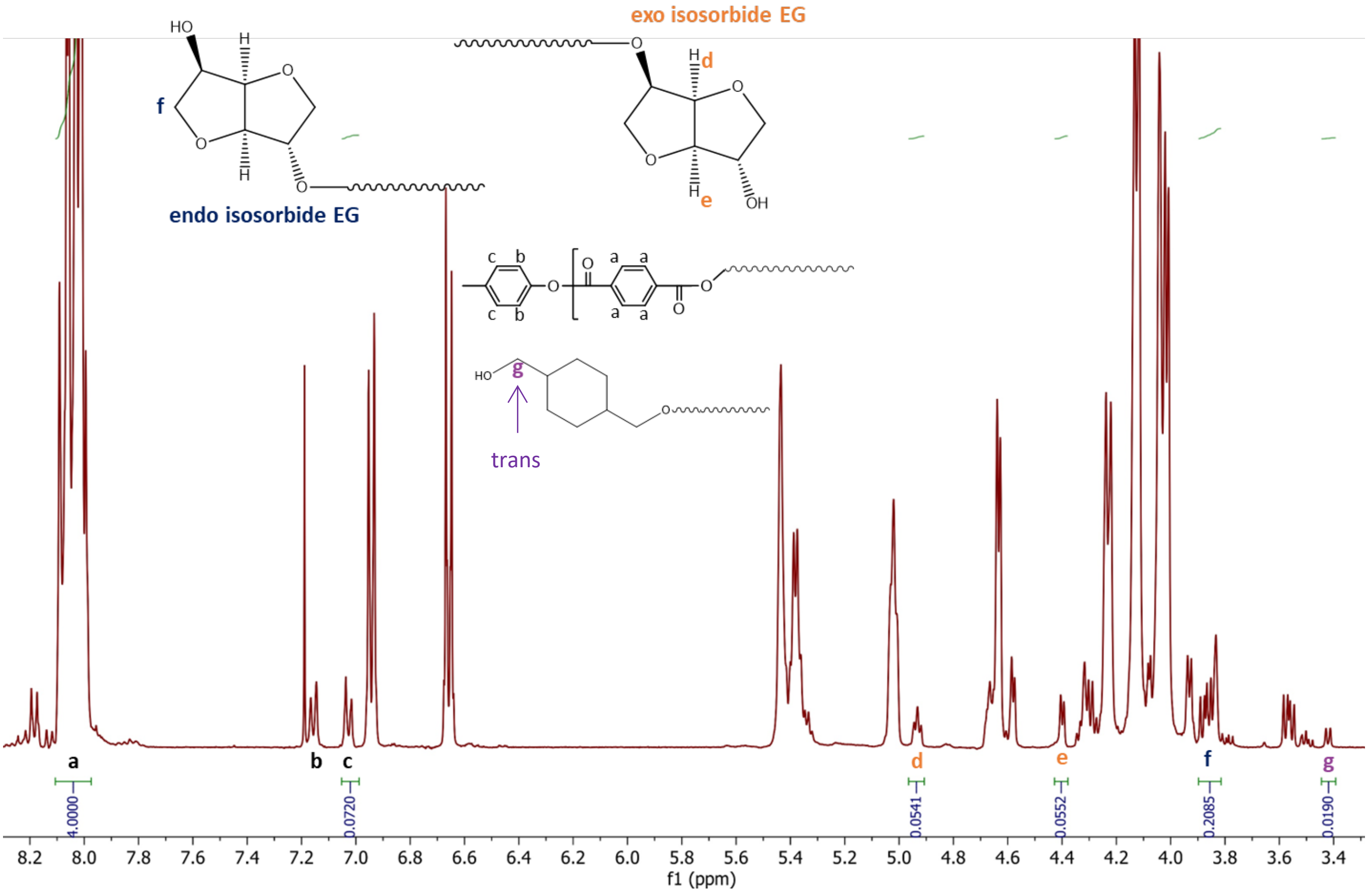

Figure S6 End groups in PICT highlighted for calculation example. Same reaction as ^{13}C-NMR spectrum, in autoclave with 0.3 equivalent of p-cresol. Current time: 18 h of esterification (pre-vacuum). The integrals are normalized in comparison to the terephthalate group at 8.1 ppm, with 4 protons.This image can be used as an example of the calculations presented in the paper: peak ‘c’ has 2 protons, so a total of 3.60 mol% of end groups of p-cresol relative to the repeat unit. Analogously, ‘d’ and ‘e’ have 1 proton each, totaling ~5.4 mol% of isosorbide endo end groups and ‘f’ has two protons, which totals ~10.4 mol% of exo end groups – summing the two values, isosorbide end groups represent ~15.8 mol% compared to the repeat unit. At last, ‘g’ has 2 protons for the trans CHDM phase (70% of total), so in total 0.95 mol%/70% = 1.36 mol% total for CHDM.

Table S1 Progress of end groups during esterification in different polymerization reactions of PICT and PICF. "Bound" indicates the amount that is attached to the chains, as opposed to unreacted (free) molecules.

System	time (h)	Bound IS (mol% vs repeat unit)	Bound CHDM (mol%)	IS EG (mol%)	CHDM EG (mol%)	Solv EG (mol%)	Solv/diol EG	total EG (mol%)	MeOH EG (mol%)	repeat unit length	M_n estimated NMR (g/mol)
PICF/$Cre^{1.2}$	2	32.1%	47.2%	20.6%	1.5%	10.6%	48.3%	32.7%	0.0%	6.1	1624.4
	4	36.2%	45.3%	14.1%	0.7%	12.6%	85.7%	27.4%	0.0%	7.3	1937.7
	6	37.6%	43.0%	11.4%	0.9%	16.7%	136.5%	28.9%	0.0%	6.9	1834.9
	7	41.6%	50.5%	11.5%	0.9%	16.5%	132.7%	28.9%	0.0%	6.9	1832.8
PICT/$Cre^{0.6}$	2	31.5%	72.3%	46.1%	8.9%	2.5%	4.5%	57.4%	0.0%	3.5	958.9
	8	49.4%	52.2%	12.6%	1.1%	7.9%	57.5%	21.6%	0.0%	9.3	2553.9
	10	49.6%	51.2%	11.8%	1.0%	8.2%	64.1%	21.0%	0.0%	9.5	2622.4
	12	50.1%	50.3%	11.8%	1.0%	8.3%	64.7%	21.1%	0.0%	9.5	2604.5
PICT/$Cre^{1.2}$ from DMT	5.5	40.4%	47.2%	23.2%	3.0%	9.3%	35.4%	54.3%	18.8%	3.7	1014.6
	11	44.2%	49.1%	21.3%	2.2%	11.9%	50.7%	46.5%	11.1%	4.3	1184.9
	15	46.0%	49.6%	20.1%	1.9%	13.7%	62.0%	41.5%	5.8%	4.8	1325.4
PICT/$MP^{1.0}$	3	35.1%	54.3%	35.7%	6.5%	8.6%	20.5%	50.8%	0.0%	3.9	1084.6
	5	40.7%	52.1%	26.9%	3.6%	12.2%	40.0%	42.6%	0.0%	4.7	1291.2
	6	42.0%	50.7%	24.6%	3.0%	14.0%	50.6%	41.6%	0.0%	4.8	1322.3
	7	43.6%	50.0%	21.1%	2.8%	15.7%	65.7%	39.6%	0.0%	5.1	1392.1
	8	44.4%	50.8%	20.9%	2.2%	17.0%	73.3%	40.1%	0.0%	5.0	1372.0
	9	45.7%	50.4%	19.9%	2.4%	17.0%	76.0%	39.3%	0.0%	5.1	1402.0
	10	46.7%	49.9%	19.1%	2.4%	18.4%	85.7%	39.9%	0.0%	5.0	1381.0
PICT from DMT	2	32.9%	49.7%	21.5%	3.2%	0.0%	0.0%	48.3%	23.6%	4.1	1140.4
	4	38.4%	50.6%	12.3%	1.3%	0.0%	0.0%	30.8%	17.3%	6.5	1787.0
	**6**	40.6%	49.5%	16.1%	2.0%	0.0%	0.0%	32.9%	14.9%	6.1	1672.4
	8	43.8%	50.6%	11.6%	1.8%	0.0%	0.0%	20.0%	6.7%	10.0	2752.8
	10	44.5%	50.8%	6.8%	0.6%	0.0%	0.0%	12.7%	5.2%	15.8	4347.2
	12	45.0%	50.4%	7.0%	0.5%	0.0%	0.0%	12.4%	4.9%	16.1	4424.7
PICT/$Cre^{1.2}$ 2 L autoclave	2	25.3%	54.1%	41.5%	20.9%	7.5%	12.1%	70.0%	0.0%	2.9	787.0
	4	34.0%	52.3%	38.8%	5.3%	9.1%	20.7%	53.3%	0.0%	3.8	1032.9
	6	39.7%	49.4%	27.9%	3.3%	10.4%	33.3%	41.5%	0.0%	4.8	1326.4
	8	42.8%	50.2%	21.0%	2.3%	13.2%	56.6%	36.4%	0.0%	5.5	1511.0
	10	44.2%	50.3%	16.3%	1.6%	15.6%	87.1%	33.5%	0.0%	6.0	1644.7
	12	50.0%	49.9%	15.9%	1.5%	17.8%	102.6%	35.2%	0.0%	5.7	1563.1
	14	47.2%	48.9%	14.8%	1.4%	17.1%	105.5%	33.3%	0.0%	6.0	1653.2
	16	47.9%	49.4%	14.1%	1.1%	17.4%	115.0%	32.5%	0.0%	6.1	1692.4
	18	47.6%	49.6%	13.4%	1.3%	18.2%	123.8%	32.9%	0.0%	6.1	1671.9
	20	47.6%	49.5%	13.9%	1.3%	17.0%	111.3%	32.2%	0.0%	6.2	1711.0

Table S2 Optimization of PICT systems regarding p-cresol quantity. This information relates to Figure 3 in the main file.

Cresol eq.	T_g (°C)	M_n (kg/mol)	full vacuum (PC) (h)	$t_{est.}$ (h)	IS excess feed (mol %)	Cre EG (pre PC) (mol %)	IS EG / CHDM EG (pre PC) (mol %)	solv EG/ diol EG (pre PC) (-)	Polymer composition IS / CHDM (mol %)
**0.00**	138.6	14.6	3.3	9.3	3%	0.0	3.8 / 0.4	0.00	49.3 / 49.0
**0.15**	142.0	19.4	2.0	14.0	3%	0.4	5.5 / 0.6	0.06	49.9 / 50.2
**0.30**	146.6	24.3	2.0	14.0	3%	3.8	9.8 / 0.9	0.36	49.9 / 49.5
**0.60**	147.0	33.2	2.0	12.0	3%	7.7	10.9 / 1.0	0.65	50.9 / 49.5
**1.20**	147.5	30.0	2.0	16.7	3%	14.8	14.8 / 1.5	0.91	50.2 / 50.3

Table S3 Conditions for reactions used for Mn vs t_{PC} time comparison (Figure 4 in main file).

Sample	M_n evolution [t_{PC}, h] (kg/mol)	t_{est} (h)	T_{est} / T_{PC} (°C)
**PICT/Cre$^{1.2}$**	41.9 [1] / 42.0 [2]	16.7	240 – 250 / 285
**PICT/Cre$^{0.6}_{(IS+3\%)}$**	23.6 [1] / 33.2 [2]	13.5	265 / 285
**PICT/Cre$^{1.2}_{(IS+3\%)}$**	27.6 [1] / 30.1 [2]	16.7	240 / 285
**PICT/Cre$^{0.3}_{(IS+3\%)}$**	23.8 [1] / 25.0 [2]	14	240 / 285
**PICT$_{(IS+10\%)}$**	16.7 [1.5] / 21.2 [2.5] / 20.5 [3] / 23.3 [4]	10	260 / 285
**PICF/Cre$^{1.2}$** **(not shown on figure 4)**	20.4 [2] / 21.9 [3] / 21.6 [4]	20	230 / 270
**PICT{DMT}$_{(IS+3\%)}$**	13.8 [1] / 14.9 [2] / 16.6 [3] / 15.8 [4] / 16.1 [5]	14	240 – 260 / 285

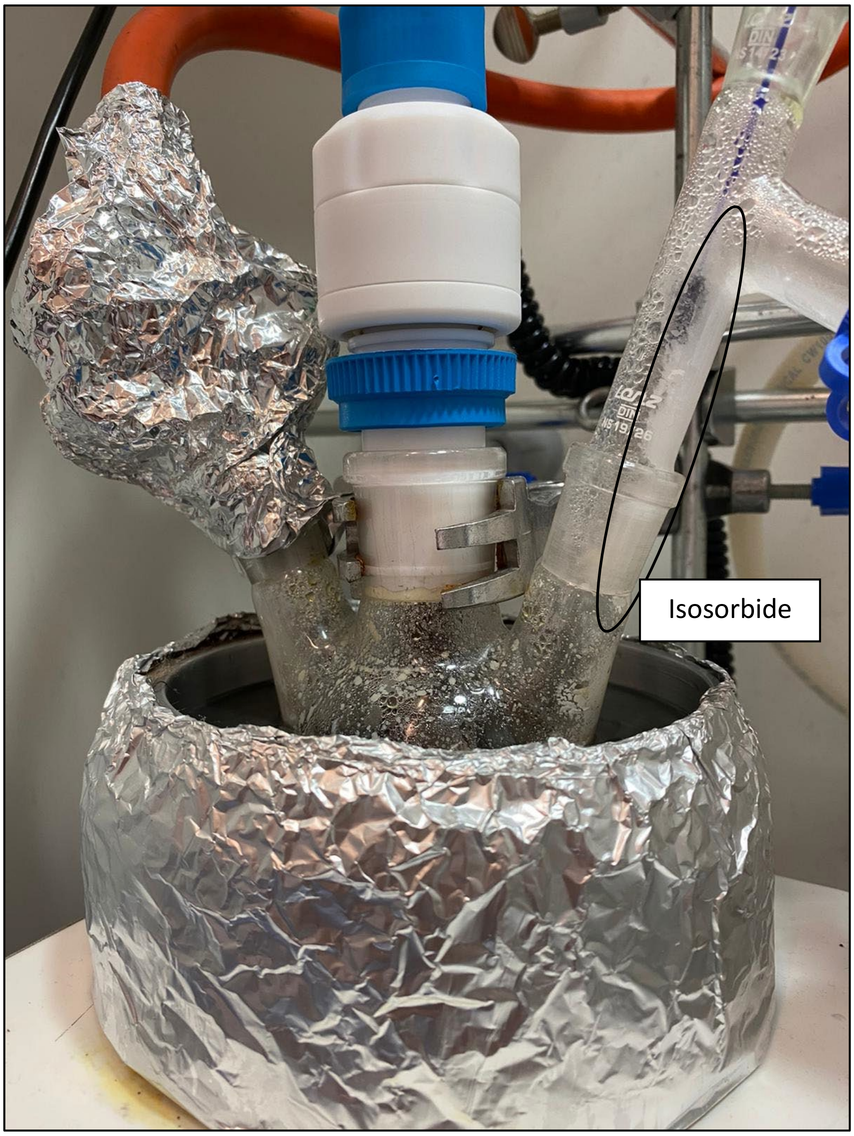

Figure S7 Sublimated/dragged isosorbide (highlighted) in a PICT system without the use of solvents

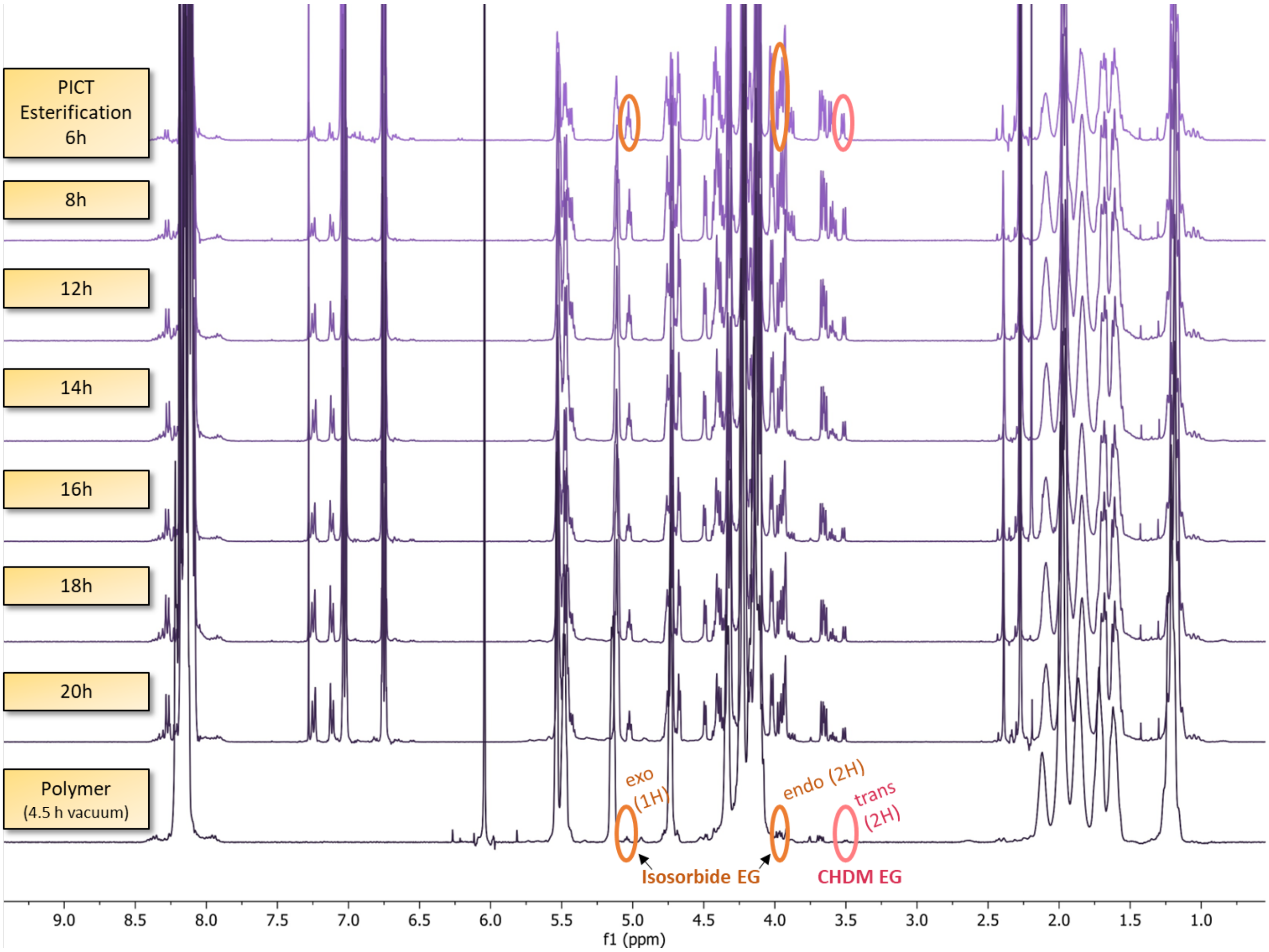

Figure S8 PICT esterification progression via proton NMR. Same reaction as the ^{13}C-NMR shows – done in a 2 L autoclave with 0.3 equivalent of p-cresol. Mono-esters (end groups) can be identified.

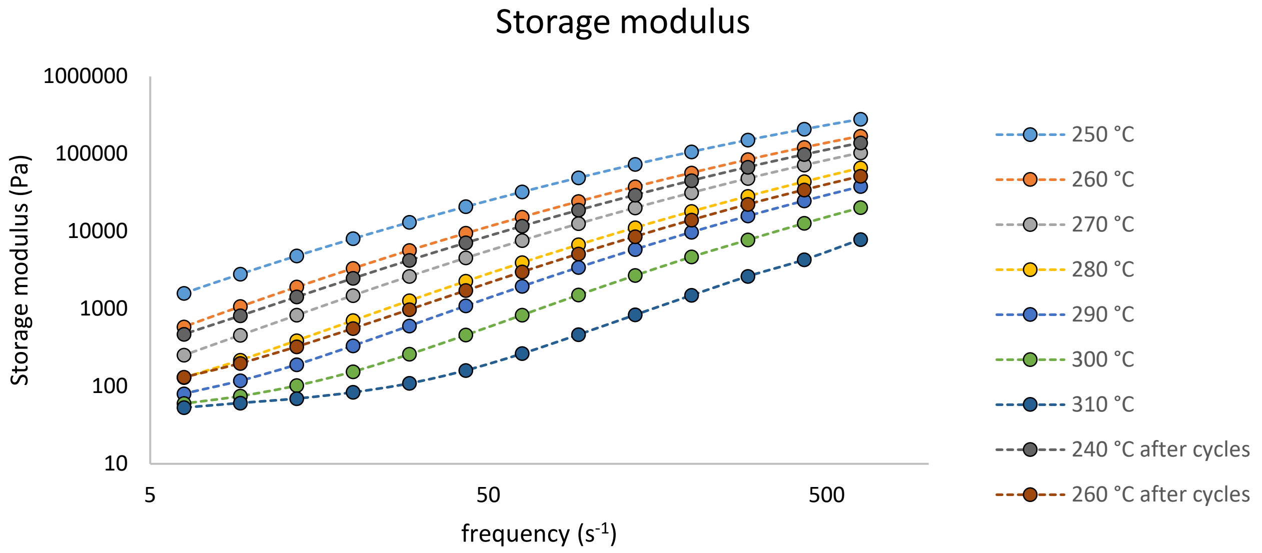

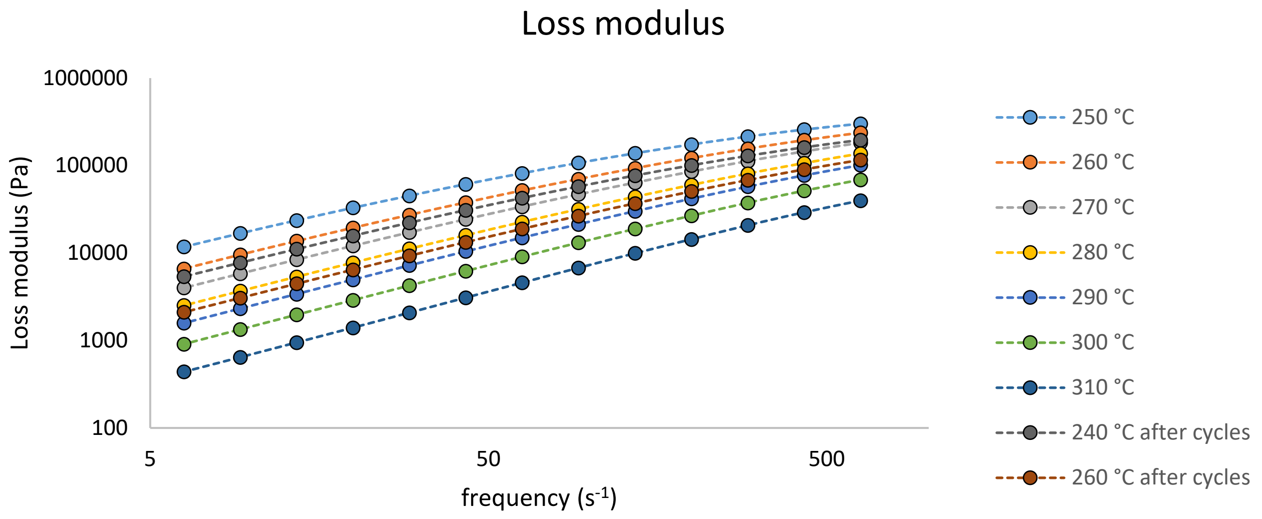

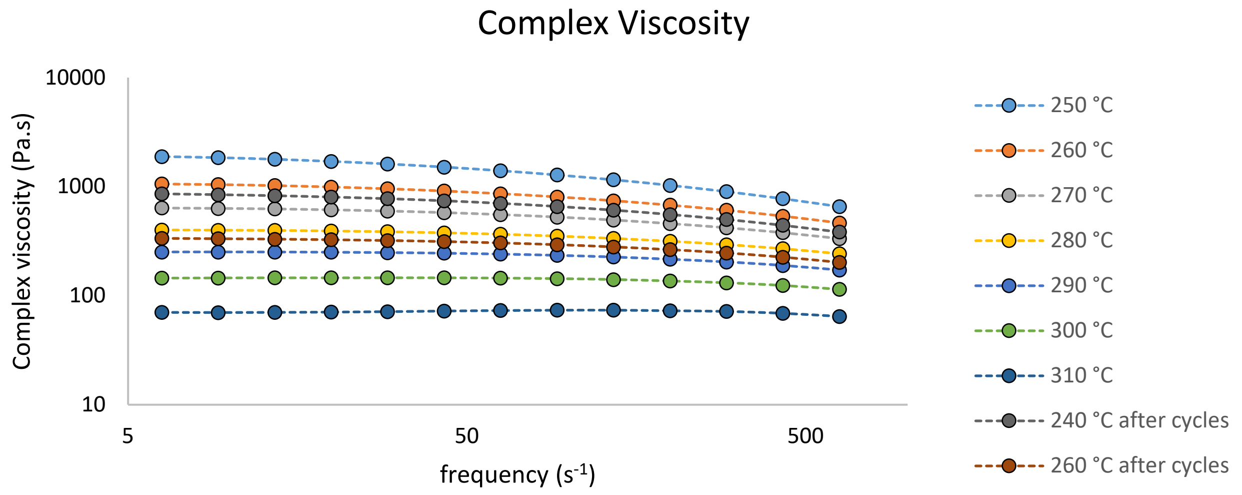

Figure S9 Storage modulus, loss modulus and complex viscosity of PICT sample produced in 2L autoclave, Mn = 16.4 kg/mol (pre-rheology test). The sample was dried in the rheometer at 180 °C for 2 hours then submitted, in sequence, to frequency sweeps at the temperatures as displayed. After the cycles (last one 310 °C), the samples were again submitted to 240 °C and 260 °C sweeps, respectively.

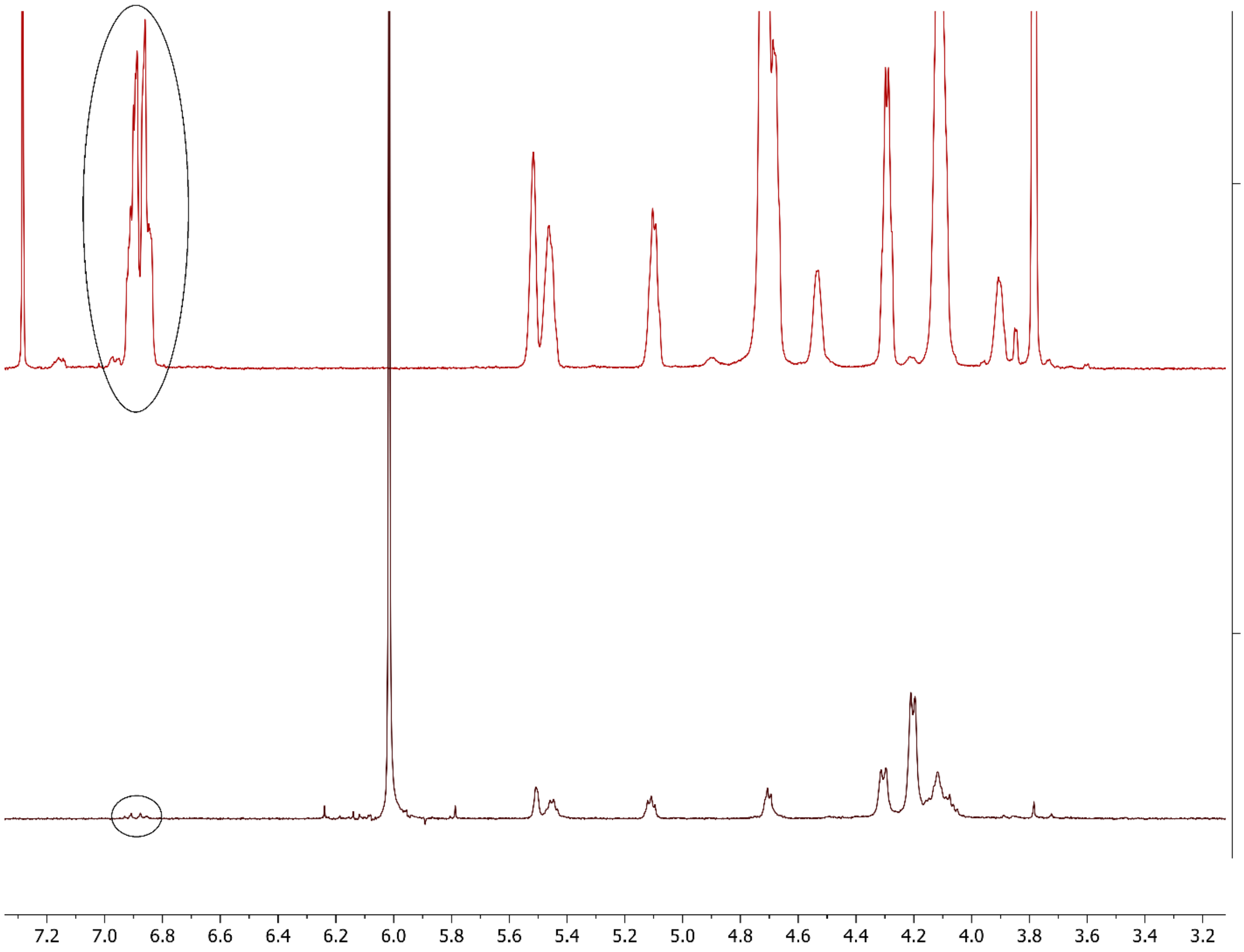

Figure S10 Two reaction systems, run in a 2-liter autoclave vessel, which presented the formation of an ether compound (highlighted) in PEIT (top) and PICT (bottom) polymerizations.

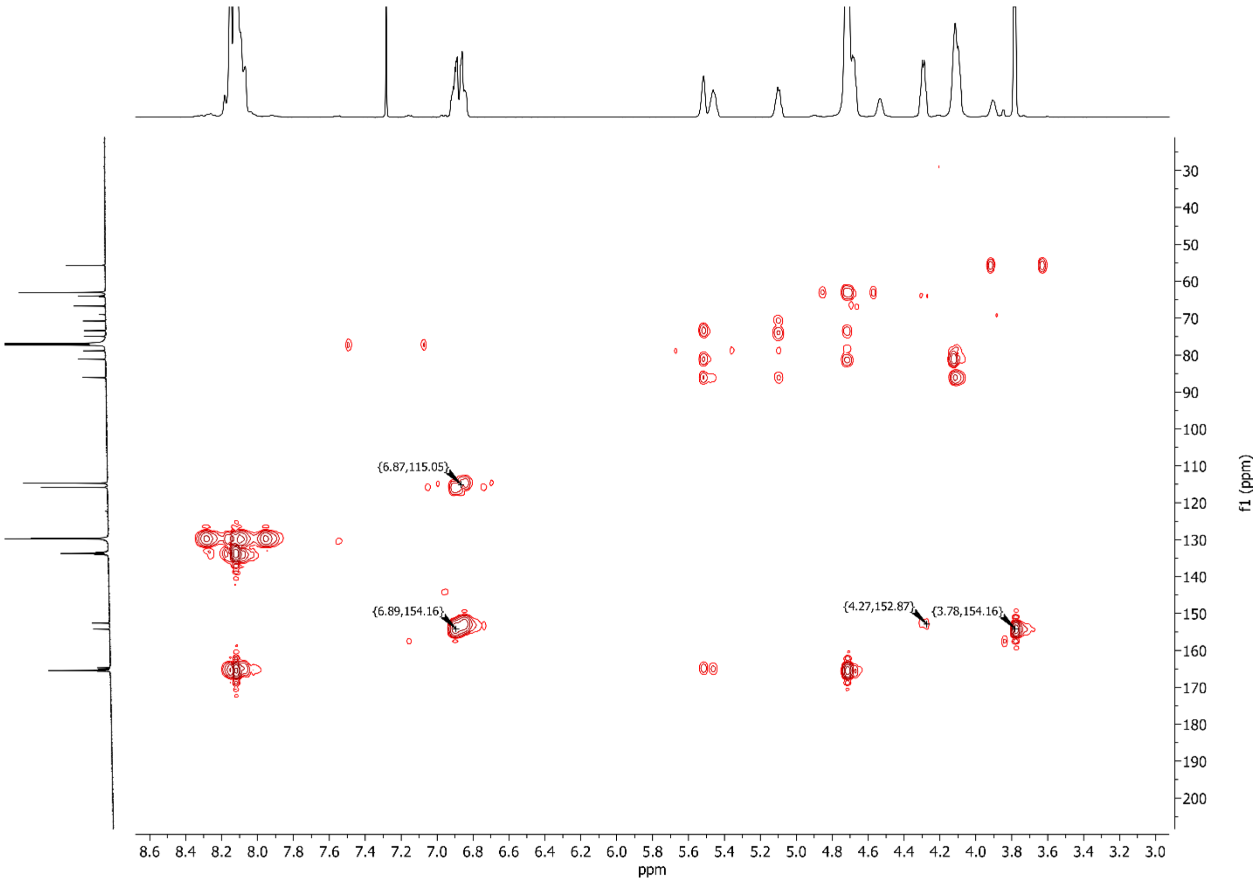

Figure S11 2D NMR (HMBC) relating an ether, formed in a PEIT system in our 2 L autoclave, with ethylene glycol correlations. Likely the ether is formed between the p-methoxyphenyl group and ethylene glycol. According to the previous figure, it is expected the same happened between p-methoxyphenyl and CHDM, however the concentrations were too low to analyze by C-NMR.

Table S4 Optimization of solvent amount using MP (4-methoxy phenol) for PICT.

Sample[a]	M_n[d] (kg/mol)	PDI	T_g (°C)
**PICT (no solv.)**	14.8	2.7	135.0
**PICT/MP[0.3]**	12.5	2.2	136.3
**PICT/MP[0.6]**	16.9	2.3	139.0
**PICT/MP[0.9]**	19.2	2.3	142.4
**PICT/MP[1.5]**	19.3	2.3	142.5

References

1 W. J. Yoon, K. S. Oh, J. M. Koo, J. R. Kim, K. J. Lee and S. S. Im, Advanced polymerization and properties of biobased high Tg polyester of isosorbide and 1,4-cyclohexanedicarboxylic acid through in situ acetylation, *Macromolecules*, 2013, **46**, 2930–2940.

2 W. J. Yoon, S. Y. Hwang, J. M. Koo, Y. J. Lee, S. U. Lee and S. S. Im, Synthesis and characteristics of a biobased high-T g terpolyester of isosorbide, ethylene glycol, and 1,4-cyclohexane dimethanol: Effect of ethylene glycol as a chain linker on polymerization, *Macromolecules*, 2013, **46**, 7219–7231.

3 S. Legrand, N. Jacquel, H. Amedro, R. Saint-Loup, J. P. Pascault, A. Rousseau and F. Fenouillot, Synthesis and properties of poly(1,4-cyclohexanedimethylene-co-isosorbide terephthalate), a biobased copolyester with high performances, *Eur. Polym. J.*, 2019, **115**, 22–29.

4 T. Amari, K. Nishimura, K. Minou, A. Kawabata and Y. Ozaki, End-group characterization of homo- and copolyesters of cyclohexane-1,4-dimethanol, *J. Polym. Sci. Part A Polym. Chem.*, 2001, **39**, 665–674.
